# Interpretable Machine Learning Model for Pulmonary Hypertension Risk Prediction: Retrospective Cohort Study

**DOI:** 10.2196/74117

**Published:** 2025-09-24

**Authors:** Hongxia Jiang, Han Gao, Dexin Wang, Qingli Zeng, Xiaojun Hao, Zhenshun Cheng

**Affiliations:** 1 Department of Respiratory and Critical Care Medicine, Zhongnan Hospital of Wuhan University, Number 169, Donghu Road, Wuchang District, Wuhan, 430000, China; 2 Department of Respiratory and Critical Care Medicine, Qichun County People's Hospital, Huanggang, China; 3 Wuhan Research Center for Infectious Diseases and Cancer, Chinese Academy of Medical Sciences, Wuhan, China; 4 Hubei Engineering Center for Infectious Disease Prevention, Control and Treatment, Wuhan, China

**Keywords:** pulmonary hypertension, machine learning, risk prediction, echocardiography, clinical data

## Abstract

**Background:**

Pulmonary hypertension (PH) is a progressive disorder characterized by elevated pulmonary artery pressure and increased pulmonary vascular resistance, ultimately leading to right heart failure. Early detection is critical for improving patient outcomes.

**Objective:**

The diagnosis of PH primarily relies on right heart catheterization, but its invasive nature significantly limits its clinical use. Echocardiography, as the most common noninvasive screening and diagnostic tool for PH, provides valuable patient information. This study aims to identify key PH predictors from echocardiographic parameters, laboratory tests, and demographic data using machine learning, ultimately constructing a predictive model to support early noninvasive diagnosis of PH.

**Methods:**

This study compiled comprehensive datasets comprising echocardiography measurements, clinical laboratory data, and fundamental demographic information from patients with PH and matched controls. The final analytical cohort consisted of 895 participants with 85 evaluated variables. Recursive feature elimination was used to select the most relevant echocardiographic variables, which were subsequently integrated into a composite ultrasound index using machine learning techniques, XGBoost (Extreme Gradient Boosting). LASSO (least absolute shrinkage and selection operator) regression was applied to select the potential predictive variable from laboratory tests. Then, the ultrasound index variables and selected laboratory tests were combined to construct a logistic regression model for the predictive diagnosis of PH. The model’s performance was rigorously evaluated using receiver operating characteristic curves, calibration plots, and decision curve analysis to ensure its clinical relevance and accuracy. Both internal and external validation were used to assess the performance of the constructed model.

**Results:**

A total of 16 echocardiographic parameters (right atrium diameter, pulmonary artery diameter, left atrium diameter, tricuspid valve reflux degree, right ventricular diameter, E/E’ [ratio of mitral valve early diastolic inflow velocity (E) to mitral annulus early diastolic velocity (E’)], interventricular septal thickness, left ventricular diameter, ascending aortic diameter, left ventricular ejection fraction, left ventricular outflow tract velocity, mitral valve reflux degree, pulmonary valve outflow velocity, mitral valve inflow velocity, aortic valve reflux degree, and left ventricular posterior wall thickness) combined with 2 laboratory biomarkers (prothrombin time activity and cystatin C) were identified as optimal predictors, forming a high-performance PH prediction model. The diagnostic model demonstrated high predictive accuracy, with an area under the receiver operating characteristic curve of 0.997 in the internal validation and 0.974 in the external validation. Both calibration plots and decision curve analysis validated the model’s predictive accuracy and clinical applicability, with optimal performance observed at higher risk stratification cutoffs.

**Conclusions:**

This model enhances early PH diagnosis through a noninvasive approach and demonstrates strong predictive accuracy. It facilitates early intervention and personalized treatment, with potential applications in broader cardiovascular disease management.

## Introduction

Pulmonary hypertension (PH) is a progressive and complex vascular disorder characterized by elevated mean pulmonary artery pressure (mPAP) and increased pulmonary vascular resistance, ultimately leading to right heart failure [1]. According to the World Symposium on Pulmonary Hypertension classification system, PH is categorized into 5 major groups. Delayed diagnosis and treatment impose an unacceptably high mortality burden across all categories of PH patients. However, the current gold standard for PH diagnosis—right heart catheterization (RHC)—is significantly limited in clinical application due to its invasive nature, resulting in delayed PH diagnosis. Thus, early identification and risk assessment through noninvasive methods are critical for improving patient outcomes [2-5].

Echocardiography, as a noninvasive imaging modality, has become integral to the evaluation of PH. It enables clinicians to assess right ventricular size and function and estimate pulmonary artery pressures through tricuspid regurgitation velocity (TRV) and pulmonary artery systolic pressure. Several studies have highlighted the importance of echocardiographic parameters including right ventricular size, function, and TRV in predicting PH severity and outcomes [6-8]. Moreover, comprehensive models such as the Registry to Evaluate Early and Long-term Pulmonary Arterial Hypertension Disease Echocardiographic Correlation Hemodynamic Observational Study score, which integrate multiple echocardiographic variables, have significantly improved the ability to predict PH risk and have been validated across multiple cohorts [6,9].

However, relying solely on echocardiographic data for PH diagnosis and risk prediction has limitations. Recent research has increasingly emphasized the need to integrate echocardiographic findings with clinical data, including age, sex, BMI, and comorbidities, to enhance predictive accuracy [10,11]. For instance, studies involving hyperthyroid patients demonstrated that models incorporating both clinical variables and echocardiographic parameters significantly improved PH risk assessment [12]. Likewise, nomogram models that integrate these data have shown efficacy in evaluating PH risk for both postoperative patients and those with concurrent conditions such as obstructive sleep apnea [13,14]

Beyond variable collection, methodological rigor is equally critical for building precise predictive models. In recent years, the application of machine learning techniques to PH risk prediction has gained traction. Methods such as recursive feature elimination (RFE) and advanced algorithms such as random forests and XGBoost (Extreme Gradient Boosting) have been shown to optimize feature selection, improving model robustness and prediction accuracy [14]. These advancements, particularly when integrating echocardiographic and clinical data, have yielded models that outperform traditional methods in PH risk prediction [5].

In this study, we systematically collected comprehensive datasets including echocardiographic parameters, laboratory test results, and baseline demographic characteristics (age and sex) from both PH patients and matched controls. Using multiple machine learning approaches, we developed an early diagnostic and predictive model for PH. The model’s performance was rigorously evaluated through receiver operating characteristic (ROC) analysis, decision curve analysis (DCA), and calibration curves, with both internal and external validation procedures. Comparative analyses against existing PH diagnostic models demonstrated the superior predictive accuracy and clinical utility of our novel model, highlighting its significant advancements in early PH detection through the integration of multimodal biomarkers and optimized machine learning architecture.

## Methods

### Ethical Considerations

This study was approved by the research ethics commission of Wuhan Zhongnan Hospital, and the requirement for informed consent was waived by the ethics commission (2023185). We have anonymized all patient identifiers, including names and hospital numbers, from the original dataset. The published information contains no data that could be used to infer the identity of individual patients. Furthermore, all participants will benefit from this research outcome through complimentary risk prediction for pulmonary arterial hypertension.

### Study Population and Data Collection

This retrospective study analyzed data from 294 patients with PH and 1231 control subjects who underwent echocardiographic evaluation at Zhongnan Hospital of Wuhan University between January 2022 and April 2024. Propensity score matching was implemented to mitigate potential confounding factors. This cohort served as both the training and internal validation dataset. For external validation, we prospectively collected an independent dataset comprising patients evaluated from May 2024 through May 2025. The inclusion criteria for the PH group were (1) a discharge diagnosis of PH; (2) confirmation of PH through RHC; and (3) participants aged >14 years, as pediatric and adult cardiac parameters have distinct reference ranges. Patients were excluded if (1) the mPAP, as measured by RHC was <20 mm Hg; or (2) the mPAP data from RHC were missing. The inclusion criteria for the control group were (1) echocardiography results indicating no abnormalities in cardiac morphology, valve function, or ventricular wall motion; and (2) age >14 years. Control subjects were excluded if more than 5% of their data were missing.

### Data Collection and Variables

Data collected included demographic information (age and gender), echocardiographic parameters, and laboratory test results. Echocardiographic parameters assessed cardiac structure and function, including measurements such as ascending aortic diameter, left atrial and left ventricular size, interventricular septal thickness, and pulmonary artery diameter (PAD). Additionally, the degree of regurgitation, flow velocity, and pressure gradients for the mitral, tricuspid, and aortic valves were recorded. All echocardiographic measurements were double-checked to ensure consistency and accuracy. Laboratory data included complete blood count, coagulation profiles, liver and kidney function, and electrolytes.

### Data Preprocessing

In the data preprocessing phase, missing values were imputed with each variable’s median using R (version 1.4.3; R Foundation) to ensure completeness. This approach was applied uniformly across both echocardiographic and laboratory variables, which had varying degrees of missingness, thereby maintaining sample size while minimizing bias from data exclusion. We ran stratified 10-fold cross-validation, limiting oversampling to the training folds to preserve unbiased evaluation in the held-out folds. Finally, continuous variables were mean-centered and scaled to unit variance, and categorical variables were encoded as factors for downstream analyses.

### Feature Selection

We used stratified sampling to randomly split the data into training and testing sets in a 3:1 ratio. For selecting features from echocardiographic variables, we applied RFE using a random forest-based approach. RFE iteratively removed features with the least contribution to model performance, ultimately identifying the optimal subset of features. To ensure robustness and generalizability, we used 10-fold cross-validation during feature selection.

Using the selected echocardiographic features, we trained multiple machine learning models, including logistic regression, LASSO (least absolute shrinkage and selection operator) regression, elastic net, decision tree, random forest, XGBoost, support vector machine, k-nearest neighbors, naive Bayes, and gradient boosting machine. Model training and evaluation were primarily conducted using the *caret* package [15]. We used 10-fold cross-validation and grid search to optimize the hyperparameters. The primary performance metric was the area under the receiver operating characteristic curve (AUC), which was computed and plotted using the *pROC* package. The model with the highest AUC was selected as the optimal model for the echocardiographic features. For the optimal model, we further performed internal validation using bootstrap methods to evaluate its robustness. To enhance model interpretability, we applied SHAP (Shapley Additive Explanations) to quantify each feature’s contribution to an individual prediction. In lay terms, a SHAP value represents how much a given variable increases or decreases the predicted PH risk for a patient compared to the average risk. Finally, we developed an ultrasound index based on the optimal model in the training and test sets.

For feature selection from routine clinical variables, we conducted LASSO regression analysis using the *glmnet* package. A 10-fold cross-validation was used to select the optimal regularization parameter. Specifically, we calculated a range of lambda values and chose lambda.1se, which is the lambda value with the best performance in cross-validation, adjusted by 1 SE.

### Logistic Regression and Nomogram Construction

After identifying the optimal echocardiographic features and selected clinical variables, we combined them to develop a comprehensive logistic regression model. The model was fitted using the “lrm” function from the *rms* package. To prevent overfitting, we incorporated L2 regularization (learning 2 ridge regression) as needed. Analysis of model coefficients provided insights into the contribution of each feature to predicting PH.

To facilitate the clinical application of the model, we constructed a nomogram using the “nomogram” function from the *rmsv* package. This nomogram visualizes the logistic regression model in an easy-to-use format, allowing clinicians to estimate an individual’s probability of developing PH by summing the scores for each predictive variable. The use of a nomogram ensures the interpretability and practicality of the model in a clinical setting.

### Model Calibration and DCA

To assess the performance of the nomogram and logistic regression model, we used the ROC curve. To evaluate the consistency between the predicted probabilities and observed outcomes, calibration curves were plotted using the “calibrate” function from the *rms* package. Additionally, DCA was conducted using the *rmda* package to quantify the net benefit of the model across different threshold probabilities, providing insight into its clinical utility. Finally, we developed a web-based tool to facilitate the prediction of the risk of PH using R packages *shiny* (version 1.9.1).

### External Validation

We externally validated the model using an independent cohort of patients admitted to the Respiratory Medicine Department at Zhongnan Hospital, Wuhan University, between May 2024 and May 2025. The inclusion and exclusion criteria were identical to those of the derivation cohort.

### Statistical Analysis

The SHAP analysis was conducted using SHAP (version 0.46.0) in Python (version 3.10.8; Python Software Foundation), while all other statistical analyses were performed using R (version 4.4.1). For continuous variables that followed a normal distribution, group comparisons were made using 2-tailed *t* tests. For continuous variables that did not follow a normal distribution, nonparametric tests, such as the Mann-Whitney *U* test or the Kruskal-Wallis test, were applied. Categorical variables were presented as percentages and compared between groups using the chi-square test. A *P*<.05 was considered statistically significant.

## Results

### Statistical Characterization and Intergroup Comparisons of Clinical Variables in Patients With PH Versus Matched Controls

This study ultimately included a cohort of 714 control participants and 181 patients with PH. As shown in Table 1, PH patients exhibited significantly enlarged ascending aorta, left atrium, left ventricle, right atrium, and right ventricle dimensions (all *P*<.05), along with reduced left ventricular ejection fraction (%), indicating progressive chamber dilation and impaired systolic function. Hematologic analysis revealed elevated red blood cell counts and neutrophil percentage, coupled with reduced platelet counts and lymphocyte percentage (all *P*<.05), suggesting a proinflammatory, hypercoagulable profile. Patients with PH had prolonged prothrombin time, reduced prothrombin time activity, and increased international normalized ratio, in addition to elevated total, direct, and indirect bilirubin levels (all *P*<.05), reflecting both coagulopathy and hepatic congestion. Serum cystatin C (CysC) is also found to be significantly elevated in patients with PH. Further, patients with PH experienced electrolyte imbalances—lower potassium, sodium, and phosphorus levels alongside higher chloride and calcium (all *P*<.05)—indicating disrupted electrolyte homeostasis. Together, these multisystem alterations underscore the complex structural, hematologic, coagulation, hepatic, and electrolyte derangements characteristic of PH.

**Table 1.** Characteristics of the analyzed cohort.

Variables		Normal (n=714)	PH^a^ (n=181)	Overall (n=895)	*P* value
Age (years)					.38
	Mean (SD)	52.0 (13.8)	53.2 (16.9)	52.3 (14.5)	
	Median (Min^b^, Max^c^)	53.0 (15.0, 88.0)	54.7 (16.0, 85.1)	54.0 (15.0, 88.0)	
Sex, n (%)					.81
	Male	310 (43.4)	81 (44.8)	391 (43.7)	
	Female	404 (56.6)	100 (55.2)	504 (56.3)	
Ascending aortic diameter (AAD, cm)					.002
	Mean (SD)	3.02 (0.280)	3.15 (0.540)	3.05 (0.352)	
	Median (Min, Max)	3.10 (2.20, 3.70)	3.20 (1.80, 4.90)	3.10 (1.80, 4.90)	
Left atrium diameter (LAD, cm)					<.001
	Mean (SD)	3.18 (0.362)	4.22 (1.18)	3.39 (0.746)	
	Median (Min, Max)	3.20 (1.80, 4.30)	4.00 (2.10, 7.70)	3.30 (1.80, 7.70)	
Left ventricular diameter (LVD, cm)					<.001
	Mean (SD)	4.41 (0.333)	4.98 (1.42)	4.53 (0.740)	
	Median (Min, Max)	4.40 (3.20, 5.30)	4.60 (2.50, 9.90)	4.40 (2.50, 9.90)	
Interventricular septal thickness (IVS, cm)					<.001
	Mean (SD)	0.933 (0.112)	0.997 (0.210)	0.946 (0.140)	
	Median (Min, Max)	0.900 (0, 1.30)	1.00 (0.500, 2.10)	0.900 (0, 2.10)	
	Missing, n (%)	0 (0)	1 (0.6)	1 (0.1)	
Left ventricular posterior wall thickness (LVPW, cm)					.002
	Mean (SD)	0.908 (0.0988)	0.968 (0.254)	0.920 (0.146)	
	Median (Min, Max)	0.900 (0.600, 1.20)	1.00 (0.500, 3.40)	0.900 (0.500, 3.40)	
	Missing, n (%)	0 (0)	1 (0.6)	1 (0.1)	
Right atrium diameter (RAD, cm)					<.001
	Mean (SD)	30.8 (3.74)	47.2 (13.1)	34.1 (9.45)	
	Median (Min, Max)	31.0 (20.0, 41.0)	46.0 (0, 107)	32.0 (0, 107)	
Right ventricular diameter (RVD, cm)					<.001
	Mean (SD)	3.01 (0.362)	4.33 (1.13)	3.28 (0.799)	
	Median (Min, Max)	3.00 (1.90, 4.10)	4.15 (2.30, 7.60)	3.10 (1.90, 7.60)	
	Missing, n (%)	0 (0)	1 (0.6)	1 (0.1)	
Pulmonary artery diameter (PAD, cm)					<.001
	Mean (SD)	20.7 (2.08)	29.8 (8.44)	22.5 (5.58)	
	Median (Min, Max)	21.0 (14.0, 31.0)	28.0 (0, 62.0)	21.0 (0, 62.0)	
Left ventricular fractional shortening (LVFS, %)					<.001
	Mean (SD)	36.0 (3.86)	32.6 (5.97)	35.4 (4.50)	
	Median (Min, Max)	35.0 (22.0, 47.0)	33.0 (12.0, 44.0)	35.0 (12.0, 47.0)	
	Missing, n (%)	0 (0)	28 (15.5)	28 (3.1)	
Left ventricular ejection fraction (LVEF, %)					<.001
	Mean (SD)	65.7 (4.54)	57.1 (14.6)	64.1 (8.34)	
	Median (Min, Max)	65.0 (53.0, 79.0)	62.0 (12.0, 75.0)	65.0 (12.0, 79.0)	
	Missing, n (%)	0 (0)	8 (4.4)	8 (0.9)	
Mitral valve inflow velocity (MV Vmax, m/s)					<.001
	Mean (SD)	0.741 (0.195)	1.01 (0.488)	0.796 (0.300)	
	Median (Min, Max)	0.700 (0.300, 1.40)	0.900 (0.300, 3.00)	0.700 (0.300, 3.00)	
	Missing, n (%)	0 (0)	3 (1.7)	3 (0.3)	
Mitral inflow A-wave peak velocity (MPAV, m/s)					.86
	Mean (SD)	0.778 (0.189)	0.782 (0.254)	0.779 (0.200)	
	Median (Min, Max)	0.800 (0.400, 1.40)	0.800 (0.300, 1.60)	0.800 (0.300, 1.60)	
	Missing, n (%)	0 (0)	52 (28.7)	52 (5.8)	
Left ventricular outflow tract velocity (LVOT, m/s)					.002
	Mean (SD)	0.912 (0.189)	0.739 (0.269)	0.906 (0.195)	
	Median (Min, Max)	0.900 (0.500, 1.80)	0.800 (0.300, 1.20)	0.900 (0.300, 1.80)	
	Missing, n (%)	0 (0)	153 (84.5)	153 (17.1)	
Aortic valve outflow velocity (AV Vmax, m/s)					.004
	Mean (SD)	1.22 (0.216)	1.32 (0.423)	1.24 (0.274)	
	Median (Min, Max)	1.20 (0.700, 2.40)	1.20 (0.500, 3.10)	1.20 (0.500, 3.10)	
	Missing, n (%)	11 (1.5)	2 (1.1)	13 (1.5)	
Pulmonary valve outflow velocity (PV Vmax, m/s)					<.001
	Mean (SD)	0.953 (0.175)	1.09 (0.458)	0.977 (0.256)	
	Median (Min, Max)	0.900 (0.600, 1.70)	1.00 (0.300, 4.10)	1.00 (0.300, 4.10)	
	Missing, n (%)	0 (0)	22 (12.2)	22 (2.5)	
Mitral valve reflux degree (MVRD, 0‐3)					<.001
	Mean (SD)	0.296 (0.457)	1.08 (1.15)	0.454 (0.729)	
	Median (Min, Max)	0 (0, 1.00)	1.00 (0, 4.00)	0 (0, 4.00)	
Aortic valve reflux degree (AVRD, 0‐3)					<.001
	Mean (SD)	0.0980 (0.298)	0.459 (0.619)	0.171 (0.411)	
	Median (Min, Max)	0 (0, 1.00)	0 (0, 3.00)	0 (0, 3.00)	
Tricuspid valve reflux degree (TVRD, 0‐3)					<.001
	Mean (SD)	0.396 (0.489)	1.85 (1.11)	0.690 (0.882)	
	Median (Min, Max)	0 (0, 1.00)	2.00 (0, 4.00)	0 (0, 4.00)	
Widening of ascending aorta, n (%)					<.001
	No	714 (100)	142 (78.5)	856 (95.6)	
	Yes	0 (0)	39 (21.5)	39 (4.4)	
Aortic valve thickening, n (%)					<.001
	No	696 (97.5)	147 (81.2)	843 (94.2)	
	Yes	18 (2.5)	34 (18.8)	52 (5.8)	
Aortic valve echo intensification, n (%)					<.001
	No	695 (97.3)	140 (77.3)	835 (93.3)	
	Yes	19 (2.7)	41 (22.7)	60 (6.7)	
Aortic valve calcification, n (%)					<.001
	No	712 (99.7)	175 (96.7)	887 (99.1)	
	Yes	2 (0.3)	6 (3.3)	8 (0.9)	
Poor closure of the aortic valve, n (%)					<.001
	No	713 (99.9)	119 (65.7)	832 (93)	
	Yes	1 (0.1)	62 (34.3)	63 (7)	
Pulmonary artery widening, n (%)					<.001
	No	714 (100)	88 (48.6)	802 (89.6)	
	Yes	0 (0)	93 (51.4)	93 (10.4)	
Poor closure of the pulmonary valve, n (%)					<.001
	No	713 (99.9)	157 (86.7)	870 (97.2)	
	Yes	1 (0.1)	24 (13.3)	25 (2.8)	
Mitral valve insufficiency, n (%)					<.001
	No	704 (98.6)	83 (45.9)	787 (87.9)	
	Yes	10 (1.4)	98 (54.1)	108 (12.1)	
Tricuspid insufficiency, n (%)					<.001
	No	677 (94.8)	33 (18.2)	710 (79.3)	
	Yes	37 (5.2)	148 (81.8)	185 (20.7)	
Ventricular septum thickened, n (%)					<.001
	No	711 (99.6)	147 (81.2)	858 (95.9)	
	Yes	3 (0.4)	34 (18.8)	37 (4.1)	
Left ventricular posterior wall thickened, n (%)					<.001
	No	714 (100)	164 (90.6)	878 (98.1)	
	Yes	0 (0)	17 (9.4)	17 (1.9)	
Atrial septal defect, n (%)					<.001
	No	714 (100)	143 (79)	857 (95.8)	
	Yes	0 (0)	38 (21)	38 (4.2)	
Ventricular septal defect, n (%)					<.001
	No	714 (100)	171 (94.5)	885 (98.9)	
	Yes	0 (0)	10 (5.5)	10 (1.1)	
E/E’^d^					<.001
	Mean (SD)	9.24 (1.99)	13.0 (5.43)	9.97 (3.33)	
	Median (Min, Max)	9.00 (4.00, 18.0)	12.0 (5.00, 33.0)	10.0 (4.00, 33.0)	
	Missing, n (%)	121 (16.9)	39 (21.5)	160 (17.9)	
WBC^e^ (10^9^/L)					.97
	Mean (SD)	6.16 (4.41)	6.15 (2.33)	6.16 (4.08)	
	Median (Min, Max)	5.67 (1.05, 103)	5.62 (2.50, 17.5)	5.65 (1.05, 103)	
	Missing, n (%)	0 (0)	1 (0.6)	1 (0.1)	
RBC^f^ (10^9^/L)					.01
	Mean (SD)	4.19 (0.660)	4.38 (0.905)	4.23 (0.720)	
	Median (Min, Max)	4.22 (1.76, 7.86)	4.27 (1.77, 8.48)	4.23 (1.76, 8.48)	
	Missing, n (%)	0 (0)	1 (0.6)	1 (0.1)	
Hb^g^ (g/L)					.14
	Mean (SD)	127 (19.4)	131 (26.1)	128 (20.9)	
	Median (Min, Max)	129 (52.8, 194)	130 (60.0, 252)	129 (52.8, 252)	
	Missing, n (%)	0 (0)	1 (0.6)	1 (0.1)	
Plt^h^ (10^9^/L)					<.001
	Mean (SD)	222 (80.4)	183 (75.3)	214 (80.8)	
	Median (Min, Max)	215 (16.0, 575)	174 (34.0, 545)	206 (16.0, 575)	
	Missing, n (%)	0 (0)	1 (0.6)	1 (0.1)	
Neutrophilic granulocyte percentage (%)					.002
	Mean (SD)	60.9 (12.4)	64.0 (11.1)	61.5 (12.2)	
	Median (Min, Max)	61.0 (12.8, 97.0)	63.6 (32.8, 94.2)	61.5 (12.8, 97.0)	
	Missing, n (%)	0 (0)	1 (0.6)	1 (0.1)	
Lymphocytes percentage (%)					.002
	Mean (SD)	28.0 (10.6)	25.3 (10.1)	27.4 (10.5)	
	Median (Min, Max)	28.0 (2.00, 72.2)	26.0 (1.70, 53.4)	27.5 (1.70, 72.2)	
	Missing, n (%)	0 (0)	1 (0.6)	1 (0.1)	
Monocyte percentage (%)					.99
	Mean (SD)	8.34 (3.94)	8.35 (2.81)	8.34 (3.74)	
	Median (Min, Max)	7.70 (0.800, 78.9)	8.20 (1.50, 19.4)	7.80 (0.800, 78.9)	
	Missing, n (%)	0 (0)	1 (0.6)	1 (0.1)	
Eosinophils percentage (%)					.13
	Mean (SD)	2.20 (2.22)	1.83 (3.11)	2.13 (2.43)	
	Median (Min, Max)	1.55 (0, 27.5)	1.20 (0, 37.0)	1.50 (0, 37.0)	
	Missing, n (%)	0 (0)	1 (0.6)	1 (0.1)	
Basophils percentage (%)					.28
	Mean (SD)	0.562 (0.375)	0.595 (0.368)	0.569 (0.373)	
	Median (Min, Max)	0.500 (0, 3.30)	0.500 (0, 2.60)	0.500 (0, 3.30)	
	Missing, n (%)	0 (0)	1 (0.6)	1 (0.1)	
Neutrophil absolute value (10^9^/L)					.30
	Mean (SD)	3.85 (2.39)	4.03 (2.03)	3.89 (2.32)	
	Median (Min, Max)	3.40 (0.200, 28.7)	3.53 (1.38, 12.9)	3.40 (0.200, 28.7)	
	Missing, n (%)	0 (0)	1 (0.6)	1 (0.1)	
Lymphocyte absolute value (10^9^/L)					.105
	Mean (SD)	1.57 (0.660)	1.48 (0.691)	1.55 (0.667)	
	Median (Min, Max)	1.50 (0.200, 6.79)	1.45 (0.180, 3.86)	1.50 (0.180, 6.79)	
	Missing, n (%)	0 (0)	1 (0.6)	1 (0.1)	
Monocyte absolute value (10^9^/L)					.47
	Mean (SD)	0.582 (3.02)	0.500 (0.248)	0.565 (2.70)	
	Median (Min, Max)	0.410 (0, 80.9)	0.470 (0.100, 2.00)	0.430 (0, 80.9)	
	Missing, n (%)	0 (0)	1 (0.6)	1 (0.1)	
Eosinophils absolute value (10^9^/L)					.33
	Mean (SD)	0.124 (0.134)	0.106 (0.242)	0.121 (0.161)	
	Median (Min, Max)	0.100 (0, 1.40)	0.100 (0, 3.04)	0.100 (0, 3.04)	
	Missing, n (%)	0 (0)	1 (0.6)	1 (0.1)	
Basophil absolute value (10^9^/L)					.03
	Mean (SD)	0.0212 (0.0326)	0.0275 (0.0341)	0.0225 (0.0330)	
	Median (Min, Max)	0 (0, 0.200)	0.0200 (0, 0.110)	0 (0, 0.200)	
	Missing, n (%)	0 (0)	1 (0.6)	1 (0.1)	
HCT^i^ (%)					.005
	Mean (SD)	38.4 (5.67)	40.1 (7.77)	38.7 (6.19)	
	Median (Min, Max)	38.8 (16.5, 61.8)	40.1 (17.6, 76.4)	38.9 (16.5, 76.4)	
	Missing, n (%)	0 (0)	1 (0.6)	1 (0.1)	
Mean RBC volume (fL)					.75
	Mean (SD)	91.8 (6.48)	92.0 (7.42)	91.9 (6.67)	
	Median (Min, Max)	92.0 (63.2, 120)	92.8 (65.5, 123)	92.1 (63.2, 123)	
	Missing, n (%)	0 (0)	1 (0.6)	1 (0.1)	
Average hemoglobin content (pg)					.03
	Mean (SD)	30.5 (2.52)	30.0 (3.08)	30.4 (2.65)	
	Median (Min, Max)	30.7 (18.1, 40.9)	30.8 (19.5, 40.3)	30.7 (18.1, 40.9)	
	Missing, n (%)	0 (0)	1 (0.6)	1 (0.1)	
Average hemoglobin concentration (g/L)					<.001
	Mean (SD)	332 (8.33)	325 (14.1)	331 (10.1)	
	Median (Min, Max)	332 (285, 364)	328 (257, 352)	332 (257, 364)	
	Missing, n (%)	0 (0)	1 (0.6)	1 (0.1)	
Erythrocyte distribution width CV^j^ (%)					<.001
	Mean (SD)	14.1 (1.94)	14.9 (2.28)	14.3 (2.04)	
	Median (Min, Max)	13.5 (11.3, 28.9)	14.3 (11.9, 26.1)	13.6 (11.3, 28.9)	
	Missing, n (%)	0 (0)	1 (0.6)	1 (0.1)	
Mean platelet volume (fL)					<.001
	Mean (SD)	8.69 (1.17)	9.69 (1.49)	8.89 (1.30)	
	Median (Min, Max)	8.50 (5.80, 14.5)	9.50 (7.10, 13.6)	8.70 (5.80, 14.5)	
	Missing, n (%)	0 (0)	6 (3.3)	6 (0.7)	
PT^k^ (s)					<.001
	Mean (SD)	11.3 (0.992)	14.7 (7.36)	12.0 (3.68)	
	Median (Min, Max)	11.1 (9.50, 19.7)	12.7 (9.30, 70.1)	11.3 (9.30, 70.1)	
	Missing, n (%)	0 (0)	1 (0.6)	1 (0.1)	
INR^l^					<.001
	Mean (SD)	1.04 (0.0920)	1.35 (0.662)	1.10 (0.332)	
	Median (Min, Max)	1.02 (0.870, 1.81)	1.16 (0.850, 6.08)	1.04 (0.850, 6.08)	
	Missing, n (%)	0 (0)	1 (0.6)	1 (0.1)	
Prothrombin time activity (%)					<.001
	Mean (SD)	96.2 (12.9)	75.3 (22.4)	92.0 (17.4)	
	Median (Min, Max)	97.0 (43.0, 130)	80.0 (10.0, 136)	94.0 (10.0, 136)	
	Missing, n (%)	0 (0)	1 (0.6)	1 (0.1)	
APTT^m^ (s)					<.001
	Mean (SD)	30.9 (3.93)	33.3 (7.91)	31.4 (5.09)	
	Median (Min, Max)	30.5 (17.4, 74.2)	31.8 (24.3, 103)	30.6 (17.4, 103)	
	Missing, n (%)	0 (0)	1 (0.6)	1 (0.1)	
TT^n^ (s)					.14
	Mean (SD)	14.5 (1.76)	16.3 (16.3)	14.9 (7.46)	
	Median (Min, Max)	14.5 (11.0, 46.7)	15.0 (12.2, 231)	14.6 (11.0, 231)	
	Missing, n (%)	0 (0)	4 (2.2)	4 (0.4)	
Fibrinogen content (mg/dL)					<.001
	Mean (SD)	328 (86.0)	297 (75.9)	322 (84.9)	
	Median (Min, Max)	314 (97.0, 743)	286 (141, 536)	308 (97.0, 743)	
	Missing, n (%)	0 (0)	2 (1.1)	2 (0.2)	
D2^o^ dimer (ng/mL)					.02
	Mean (SD)	401 (1130)	1420 (5630)	602 (2720)	
	Median (Min, Max)	136 (0, 15,900)	189 (1.00, 63,200)	141 (0, 63,200)	
	Missing, n (%)	0 (0)	5 (2.8)	5 (0.6)	
ALT^p^ (U/L)					.09
	Mean (SD)	26.0 (45.1)	84.0 (394)	35.2 (163)	
	Median (Min, Max)	18.0 (3.00, 867)	19.5 (1.00, 3330)	18.0 (1.00, 3330)	
	Missing, n (%)	0 (0)	47 (26)	47 (5.3)	
AST^q^ (U/L)					.09
	Mean (SD)	24.6 (33.2)	95.0 (556)	38.7 (252)	
	Median (Min, Max)	19.0 (4.00, 574)	22.0 (9.00, 6160)	20.0 (4.00, 6160)	
	Missing, n (%)	0 (0)	2 (1.1)	2 (0.2)	
AST/ALT					<.001
	Mean (SD)	1.22 (0.611)	8.42 (16.9)	2.65 (8.06)	
	Median (Min, Max)	1.11 (0.270, 6.86)	1.44 (0.350, 133)	1.17 (0.270, 133)	
	Missing, n (%)	0 (0)	4 (2.2)	4 (0.4)	
TBIL^r^ (μmol/L)					.003
	Mean (SD)	12.4 (12.4)	16.4 (17.0)	13.2 (13.6)	
	Median (Min, Max)	10.6 (1.50, 275)	12.0 (0.540, 103)	10.6 (0.540, 275)	
	Missing, n (%)	0 (0)	2 (1.1)	2 (0.2)	
DBIL^s^ (μmol/L)					<.001
	Mean (SD)	4.36 (12.3)	8.86 (8.70)	5.26 (11.8)	
	Median (Min, Max)	2.70 (0.300, 231)	5.60 (0.600, 51.1)	2.90 (0.300, 231)	
	Missing, n (%)	0 (0)	2 (1.1)	2 (0.2)	
IDBIL^t^ (μmol/L)					<.001
	Mean (SD)	8.66 (5.09)	12.3 (11.1)	9.39 (6.90)	
	Median (Min, Max)	7.70 (0.800, 44.1)	9.40 (1.10, 94.1)	7.90 (0.800, 94.1)	
	Missing, n (%)	0 (0)	2 (1.1)	2 (0.2)	
Total protein (g/L)					<.001
	Mean (SD)	68.6 (9.60)	54.3 (25.4)	65.7 (15.3)	
	Median (Min, Max)	68.9 (3.04, 105)	64.9 (3.20, 88.5)	68.2 (3.04, 105)	
	Missing, n (%)	0 (0)	2 (1.1)	2 (0.2)	
Alb^u^ (g/L)					<.001
	Mean (SD)	40.9 (5.48)	45.9 (12.9)	41.9 (7.82)	
	Median (Min, Max)	40.9 (15.7, 76.5)	41.5 (22.1, 86.5)	41.0 (15.7, 86.5)	
	Missing, n (%)	0 (0)	2 (1.1)	2 (0.2)	
Glb^v^ (g/L)					.70
	Mean (SD)	31.0 (26.0)	30.6 (6.03)	31.0 (23.4)	
	Median (Min, Max)	28.3 (11.7, 319)	30.1 (16.3, 47.0)	28.7 (11.7, 319)	
	Missing, n (%)	0 (0)	2 (1.1)	2 (0.2)	
Alb or Glb					<.001
	Mean (SD)	1.71 (2.31)	8.35 (12.7)	3.04 (6.61)	
	Median (Min, Max)	1.46 (0.450, 28.6)	1.51 (0.730, 48.9)	1.46 (0.450, 48.9)	
	Missing, n (%)	0 (0)	2 (1.1)	2 (0.2)	
γGGT^w^ (U/L)					.27
	Mean (SD)	37.8 (62.7)	44.9 (79.8)	39.3 (66.5)	
	Median (Min, Max)	21.5 (0.630, 927)	23.0 (0.600, 752)	22.0 (0.600, 927)	
	Missing, n (%)	0 (0)	2 (1.1)	2 (0.2)	
ALP^x^ (U/L)					.17
	Mean (SD)	80.3 (53.7)	73.1 (63.9)	78.9 (56.0)	
	Median (Min, Max)	72.0 (3.59, 612)	64.0 (11.0, 768)	70.0 (3.59, 768)	
	Missing, n (%)	0 (0)	2 (1.1)	2 (0.2)	
TBA^y^ (μmol/L)					<.001
	Mean (SD)	6.84 (22.1)	26.1 (37.4)	10.7 (27.0)	
	Median (Min, Max)	3.10 (0.100, 303)	6.60 (0.400, 204)	3.50 (0.100, 303)	
	Missing, n (%)	0 (0)	2 (1.1)	2 (0.2)	
BUN^z^ (μmol/L)					.28
	Mean (SD)	6.58 (10.0)	7.44 (9.33)	6.75 (9.91)	
	Median (Min, Max)	5.14 (1.20, 105)	5.82 (0.400, 112)	5.25 (0.400, 112)	
	Missing, n (%)	0 (0)	3 (1.7)	3 (0.3)	
Creatinine (μmol/L)					<.001
	Mean (SD)	80.9 (104)	62.0 (43.5)	77.1 (95.6)	
	Median (Min, Max)	65.0 (2.23, 1140)	62.6 (1.95, 255)	64.2 (1.95, 1140)	
	Missing, n (%)	0 (0)	4 (2.2)	4 (0.4)	
Uric acid (μmol/L)					.80
	Mean (SD)	327 (103)	331 (193)	328 (126)	
	Median (Min, Max)	317 (0.760, 839)	335 (44.8, 1000)	321 (0.760, 1000)	
	Missing, n (%)	0 (0)	4 (2.2)	4 (0.4)	
Carbon dioxide (mmol/L)					<.001
	Mean (SD)	23.9 (4.21)	110 (171)	41.0 (83.5)	
	Median (Min, Max)	23.8 (1.00, 34.9)	24.8 (12.3, 842)	23.9 (1.00, 842)	
	Missing, n (%)	0 (0)	4 (2.2)	4 (0.4)	
Serum cystatin C (mg/L)					<.001
	Mean (SD)	1.04 (0.794)	6.99 (10.5)	2.18 (5.20)	
	Median (Min, Max)	0.900 (0.360, 7.59)	1.11 (0.510, 38.1)	0.910 (0.360, 38.1)	
	Missing, n (%)	0 (0)	12 (6.6)	12 (1.3)	
Potassium (mmol/L)					<.001
	Mean (SD)	3.94 (0.462)	3.42 (1.22)	3.84 (0.707)	
	Median (Min, Max)	3.94 (0.990, 6.27)	3.84 (0.720, 5.72)	3.93 (0.720, 6.27)	
	Missing, n (%)	0 (0)	10 (5.5)	10 (1.1)	
Sodium (mmol/L)					<.001
	Mean (SD)	138 (13.9)	108 (57.5)	132 (30.8)	
	Median (Min, Max)	140 (0.940, 148)	138 (2.93, 155)	140 (0.940, 155)	
	Missing, n (%)	0 (0)	6 (3.3)	6 (0.7)	
Chlorine (mmol/L)					<.001
	Mean (SD)	103 (11.7)	113 (15.0)	105 (13.0)	
	Median (Min, Max)	105 (2.22, 114)	106 (91.0, 146)	105 (2.22, 146)	
	Missing, n (%)	0 (0)	6 (3.3)	6 (0.7)	
Calcium (mmol/L)					<.001
	Mean (SD)	2.57 (5.43)	26.0 (43.1)	7.19 (21.8)	
	Median (Min, Max)	2.30 (0.390, 105)	2.32 (1.78, 111)	2.30 (0.390, 111)	
	Missing, n (%)	0 (0)	6 (3.3)	6 (0.7)	
Magnesium (mmol/L)					.22
	Mean (SD)	2.40 (26.1)	1.20 (0.596)	2.17 (23.5)	
	Median (Min, Max)	0.880 (0.380, 667)	0.890 (0.530, 2.54)	0.880 (0.380, 667)	
	Missing, n (%)	0 (0)	14 (7.7)	14 (1.6)	
Phosphorus (mmol/L)					.01
	Mean (SD)	5.38 (45.6)	1.16 (0.281)	4.57 (41.0)	
	Median (Min, Max)	1.18 (0.310, 784)	1.17 (0.660, 2.32)	1.17 (0.310, 784)	
	Missing, n (%)	0 (0)	11 (6.1)	11 (1.2)	

a PH: pulmonary hypertension.

b Min: minimum.

c Max: maximum.

d E/E’: ratio of mitral valve early diastolic inflow velocity (E) to mitral annulus early diastolic velocity (E’).

e WBC: white blood cell.

f RBC: red blood cell.

g Hb: hemoglobin.

h Plt: platelet count.

i HCT: hematocrit.

j CV: coefficient of variation.

k PT: prolonged prothrombin time.

l INR: international normalized ratio.

m APTT: activated partial thromboplastin time.

n TT: thrombin time.

o D2: D-dimer.

p ALT: alanine aminotransferase.

q AST: aspartate aminotransferase.

r TBIL: total bilirubin.

s DBIL: direct bilirubin.

t IDBIL: indirect bilirubin.

u Alb: albumin.

v Glb: globulin.

w γGGT: gamma-glutamyl transferase.

x ALP: alkaline phosphatase.

y TBA: total bile acids.

z BUN: blood urea nitrogen.

### Systematic Selection of Key Echocardiographic Parameters to Construct an Ultrasound Index for PH Prediction

**Figure 1.**
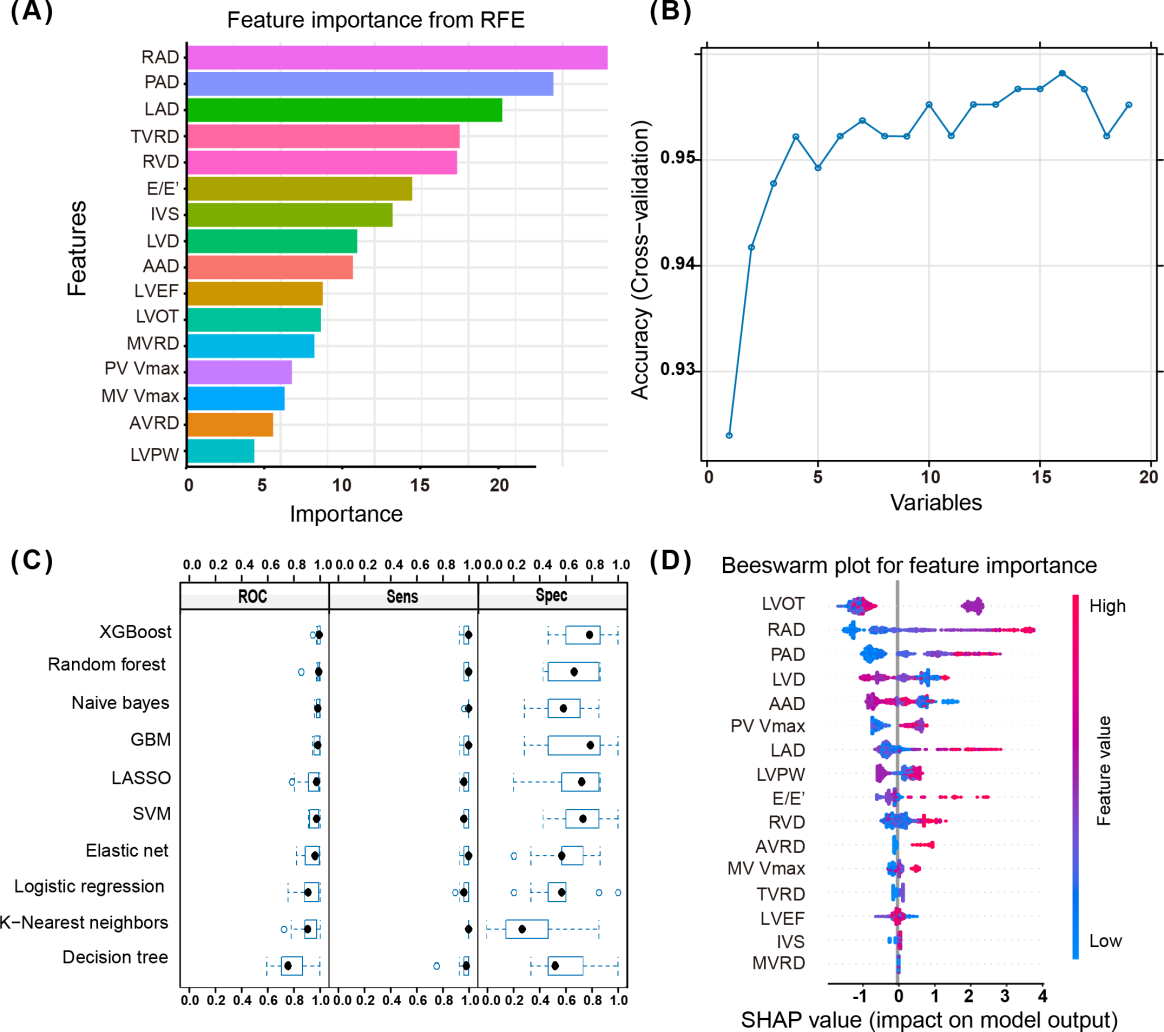
Identify important potential predictive variables for PH from echocardiographic parameters to form an ultrasound index. (**A**) Selected features from echocardiographic parameters. (**B**) Model accuracy with varying numbers of input variables. (**C**) ROC curves, sensitivity, and specificity of different machine learning algorithms. (**D**) Beeswarm plot for the feature importance of the selected echo features. AAD: ascending aortic diameter; AVRD: aortic valve reflux degree; E/E’: ratio of mitral valve early diastolic inflow velocity (E) to mitral annulus early diastolic velocity (E’); GBM: gradient boosting machine; IVS: interventricular septal thickness; LAD: left atrium diameter; LASSO: least absolute shrinkage and selection operator; LVD: left ventricular diameter; LVEF: left ventricular ejection fraction; LVOT: left ventricular outflow tract velocity; LVPW: left ventricular posterior wall thickness; MV Vmax: mitral valve inflow velocity; MVRD: mitral valve reflux degree; PAD: pulmonary artery diameter; PH: pulmonary hypertension; PV Vmax: pulmonary valve outflow velocity; RAD: right atrium diameter; RFE: recursive feature elimination; ROC: receiver operating characteristic; RVD: right ventricular diameter; Sens: sensitivity; SHAP: Shapley Additive Explanations; Spec: specificity; SVM: support vector machine; TVRD: tricuspid valve reflux degree; XGBoost: Extreme Gradient Boosting.

As shown in Figure 1A, RFE with a random forest model and 10-fold cross-validation identified 16 echocardiographic variables that maximize predictive performance, with right atrial diameter and PAD yielding the highest importance scores. Figure 1B plots cross-validation accuracy against the number of features and demonstrates optimal performance when incorporating 16 variables. Figure 1C illustrates that the XGBoost model outperformed others, excelling in AUC, sensitivity, and specificity, demonstrating high robustness in distinguishing between false positives and negatives. Figure 1D highlights SHAP analysis, which shows how each feature shifts the risk prediction for individual patients: positive SHAP values indicate increased risk, while negative values indicate decreased risk. In our model, left ventricular outflow tract velocity, right atrium diameter, and PAD produced the largest SHAP values, reflecting their strong influence on PH classification. In summary, we identified and incorporated 16 key echocardiographic parameters into a composite ultrasound index as potential predictive variables for PH.

**Figure 2.**
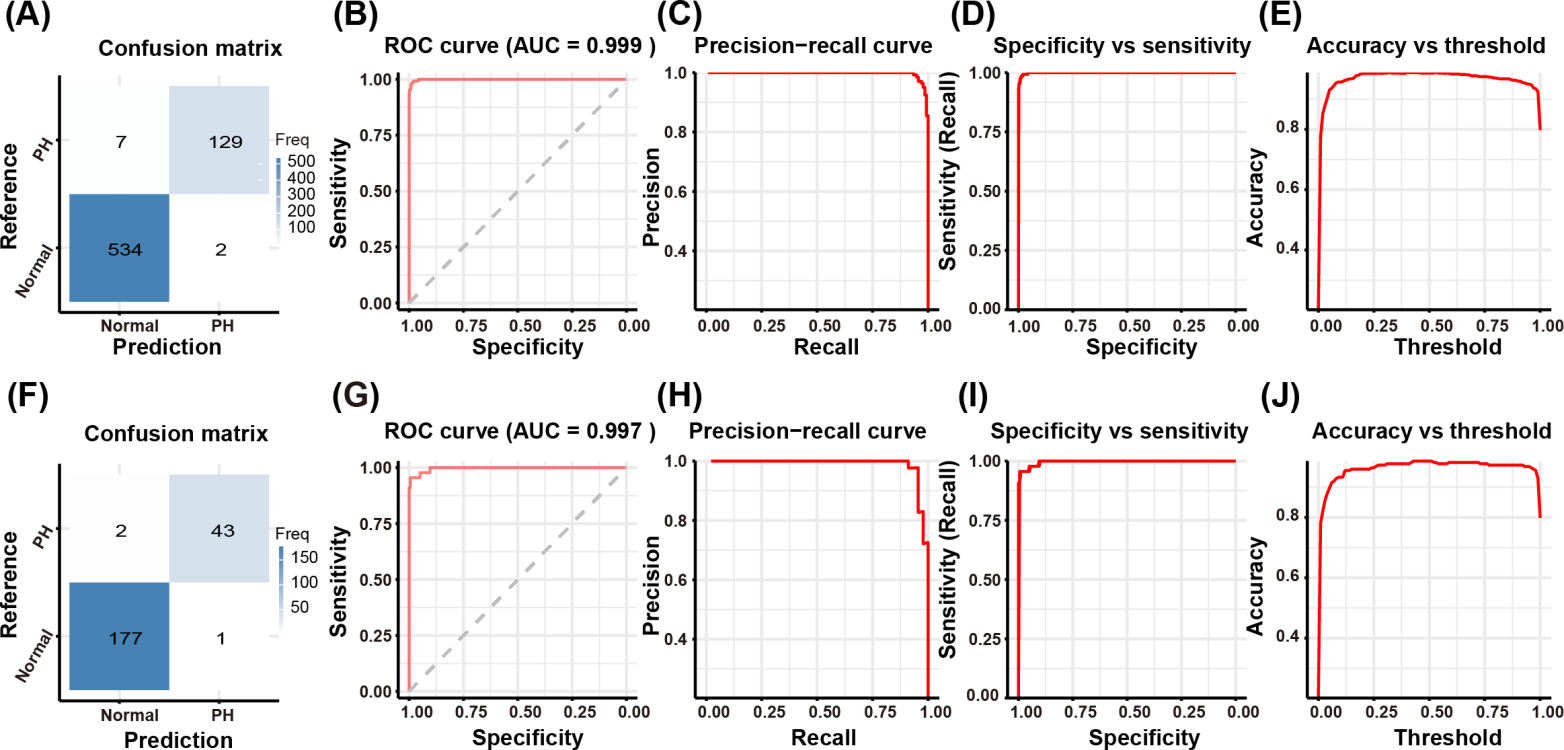
Evaluation of the predictive performance of the ultrasound index for PH in the training set and internal validation set. (A) Confusion matrix for training cohort. (B) ROC curve for training cohort (AUC=0.999). (C) Precision-recall curve for the training cohort. (D) Specificity versus sensitivity for the training cohort. (E) Accuracy versus threshold for the training cohort. (F) Confusion matrix for the validation cohort. (G) ROC curve for the validation cohort (AUC=0.997). (H) Precision-recall curve for the validation cohort. (I) Specificity versus sensitivity for the validation cohort. (J) Accuracy versus threshold for the validation cohort. AUC: area under the receiver operating characteristic curve; PH: pulmonary hypertension; ROC: receiver operating characteristic.

Figure 2A-E demonstrates the outstanding predictive performance of the ultrasound index model for PH detection, while Figure 2F-J presents the internal validation results through bootstrap resampling. Figure 2A presents the confusion matrix and ROC analysis of the ultrasound index model for PH prediction. The confusion matrix demonstrates excellent classification accuracy, with minimal misclassification between normal and PH cases. The ROC curve achieves near-perfect discrimination (AUC=0.999; Figure 2B), supported by precision-recall curves (Figure 2C) showing high positive predictive value across all sensitivity thresholds. Specificity-sensitivity analysis (Figure 2D) and accuracy-threshold analysis (Figure 2E) reveal balanced performance at the optimal diagnostic threshold, indicating robust clinical applicability. Figure 2F displays the bootstrap validation outcomes of the ultrasound index model, confirming its statistical reliability. The ROC curve maintains exceptional performance (AUC=0.997; Figure 2G) across 1000 resampled iterations, with tight CIs indicating stable predictive ability. Precision-recall (Figure 2H), specificity-sensitivity (Figure 2I), and accuracy-threshold (Figure 2J) curves show reproducible performance characteristics within narrow variability ranges (<1% fluctuation), demonstrating the model’s resistance to overfitting and dataset sampling bias.

### Development and Presentation of the Final PH Prediction Model

**Figure 3.**
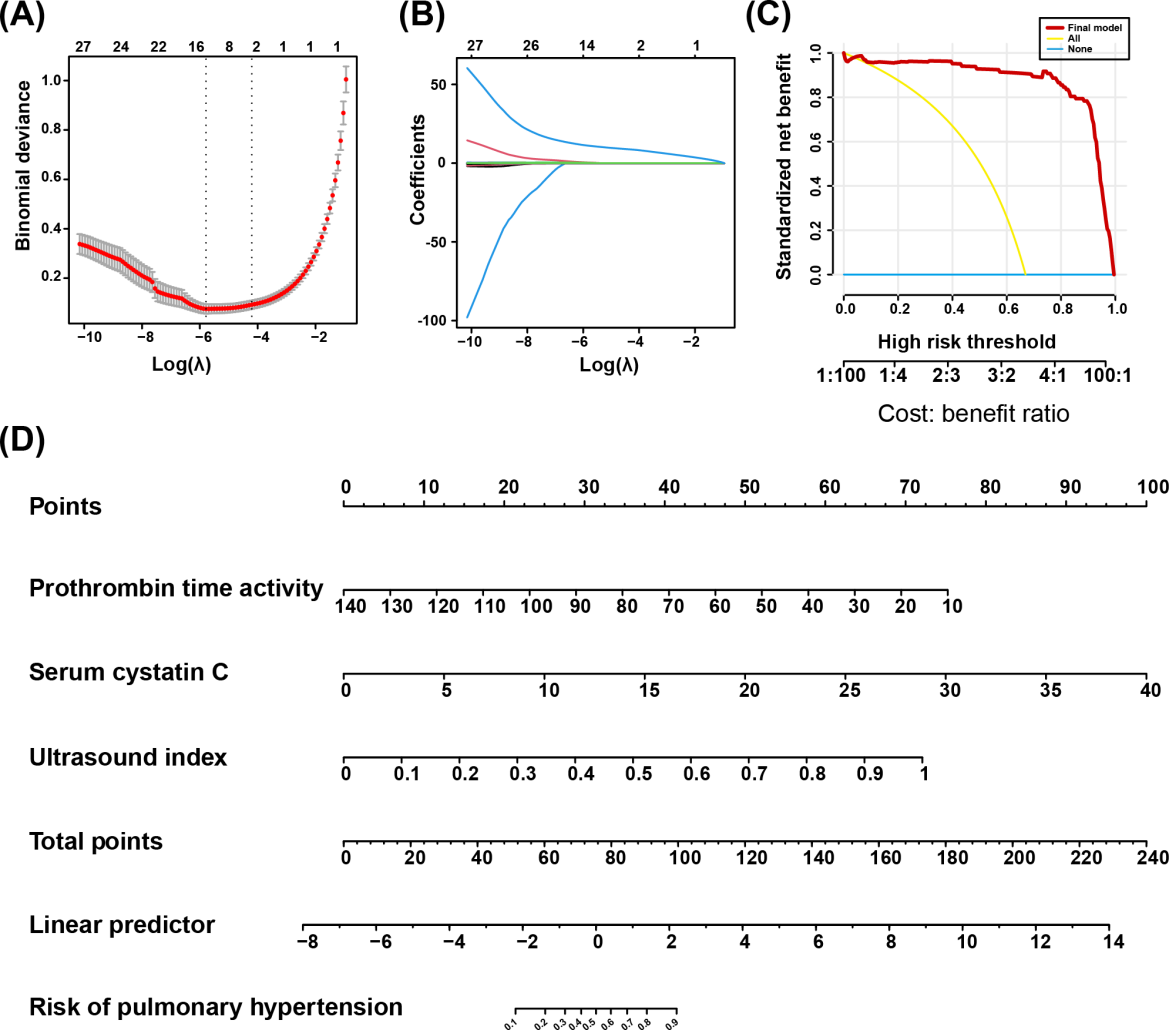
Identify key laboratory biomarkers and develop the final predictive model for PH. (A) Deviance plot from LASSO regression to select key laboratory biomarkers. (B) Coefficient path for LASSO regression to select key laboratory biomarkers. (C) Decision curve analysis of the final predictive model of PH. (D) Nomogram of the final PH prediction model. LASSO: least absolute shrinkage and selection operator; PH: pulmonary hypertension.

As shown in Figure 3A and B, LASSO regression identified 2 key clinical features—prothrombin time activity and serum CysC as the most influential variables, indicating that these variables are critical potential predictive parameters of PH. Then, we combined prothrombin time activity, serum CysC, and previously selected ultrasound index to form the final PH prediction model through logistic regression. Figure 3C demonstrates through DCA that our PH prediction model provides superior clinical net benefit compared to both “treat-all” and “treat-none” strategies across clinically relevant probability thresholds. As depicted in Figure 3D, our nomogram translates each predictor’s regression coefficient into a point scale and uses the sum of points to compute an individualized PH risk probability via a linear predictor, providing clinicians with an intuitive, quantitative tool for PH risk assessment. The nomogram translates clinical variables—including prothrombin time activity (10‐140 s mapped to 75‐0 points), serum CysC (0‐40 mg/L mapped to 0‐100 points), and the ultrasound index (0‐1 mapped to 0‐72.5 points)—into a cumulative point total. The total score is then read against the bottom probability scale to yield an individualized PH risk, providing a convenient tool that converts routine laboratory and imaging data into a directly actionable prediction of PH.

### Evaluation and Validation of the Final PH Prediction Model

**Figure 4.**
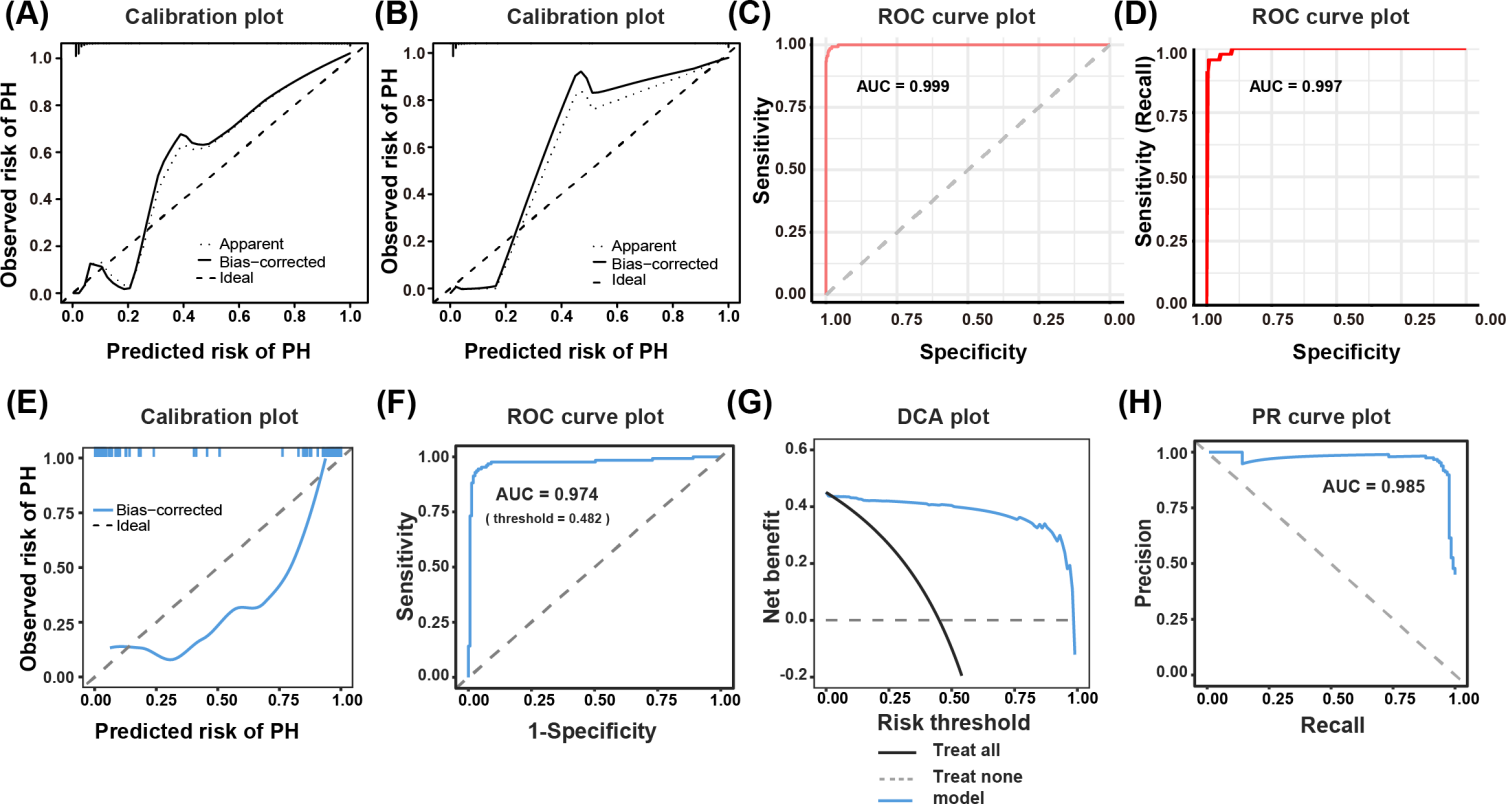
Calibration curves and ROC analysis of the final PH prediction model (nomogram) in the training, internal validation, and external validation sets. (A) Calibration curve of the nomogram in the training cohort. (B) Calibration curve of the nomogram in the internal validation cohort. (C) ROC of the nomogram in the training cohort. (D) ROC of the nomogram in the internal validation cohort. (E) Calibration curve of the nomogram in the external validation cohort. (F) ROC of the nomogram in the external validation cohort. (G) DCA plot of the nomogram in the external validation cohort. (H) PR curve plot of the nomogram in the external validation cohort. AUC: area under the receiver operating characteristic curve; DCA: decision curve analysis; PH: pulmonary hypertension; PR: precision-recall; ROC: receiver operating characteristic.

Data from different time periods were collected for external validation, including 126 patients with PH and 155 controls, with detailed information presented in Multimedia Appendix 1. Figure 4A and C present the model performance evaluation results, while Figure 4B and D display the stability analysis of internal validation (bootstrap=1000 iterations), and Figure 4E-H systematically report the external validation data. Calibration curve analysis demonstrates that the predictive model maintains excellent calibration in both the training set (Figure 4A), internal validation (Figure 4B), and external validation cohorts (Figure 4E). ROC analysis further reveals that the model exhibits stable discriminative ability across the training set (AUC=0.999, Figure 4C), internal validation (AUC=0.987, Figure 4D), and external validation (AUC=0.974, Figure 4F). In external validation, DCA shows significant net benefit across the 10%‐90% risk threshold range (Figure 4G), while the area under the precision-recall curve (AUC=0.985) confirms the model’s balanced advantage of both high precision and recall for positive cases (Figure 4H). These analytical results demonstrate that our model exhibits not only excellent predictive performance for PH, but also outstanding stability and significant clinical utility.

### Key Advantages and Innovations of Our Model Versus Current PH Predictive Models

Although predictive models for PH currently exist, these tools face significant limitations: the REVEAL score requires invasive data for optimal use and lacks imaging integration, while guideline-based echocardiography suffers from subjective interpretation variability and low sensitivity (AUC=0.70‐0.82). Both systems fail to capture early preclinical signs and the mechanistic pathways addressed by our biomarker-enhanced approach.

Our novel PH prediction model demonstrates significant advancements over existing approaches (Table 2) by integrating 16 echocardiographic parameters with 2 clinically accessible biomarkers (prothrombin time activity and CysC), achieving superior discriminative performance (AUC=0.974‐0.999 versus 0.70‐0.85 in conventional models). Unlike the REVEAL score’s reliance on invasive hemodynamics or guideline-based echocardiography’s limited echo variables, our model provides: (1) comprehensive pathophysiological insight through multimodal biomarkers reflecting coagulation and cardiac dysfunction; (2) granular risk quantification via a 240-point nomogram, enabling precise stratification; and (3) practical clinical utility through Electronic Health Record (EHR)–compatible automation. In summary, our model demonstrates significant advancements and innovations compared to existing models.

**Table 2.** Comparison of our model with existing PH^a^ risk assessment tools.

Feature	Our model	REVEAL^b^ score	Guideline-based echocardiography
Variables used	16 echocardiographic and 2 clinical (prothrombin time activity and cystatin C)	Primarily clinical (6MWD^c^, functional class, and hemodynamics)	Limited echo parameters (TR^d^ velocity and RV^e^ size)
AUC^f^	0.974‐0.999	0.79‐0.85	0.70‐0.82
Strengths	Multimodal integration (imaging and biomarkers) Objective scoring system Higher discriminative ability	Established registry data Validated long-term outcomes	Widely available First-line screening
Key advantages	Comprehensive echo assessment (16 parameters, including novel metrics such as LVOT^g^ velocity) Novel biomarkers (cystatin C adds renal or PH pathobiology dimension) Quantitative nomogram (precise risk stratification)	Lacks imaging details Requires invasive data for the full version	Subjective interpretation Limited accuracy
Clinical utility	EHR^h^-integratable Identifies early-stage PH Guides RHC^i^ decisions	Mainly for prognosis Less useful for initial diagnosis	Screening only High false-positive rate
Population	Broad applicability (includes preclinical signs)	Established patients with PH	Symptomatic suspects

a PH: pulmonary hypertension.

b REVEAL: Registry to Verify Early and Long-Term Pulmonary Arterial Hypertension Disease Management.

c 6MWD: 6-minute walk distance.

d TR: tricuspid regurgitation.

e RV: right ventricular.

f AUC: area under the receiver operating characteristic curve.

g LVOT: left ventricular outflow tract velocity.

h EHR: electronic health record.

i RHC: right heart catheterization.

**Figure 5.**
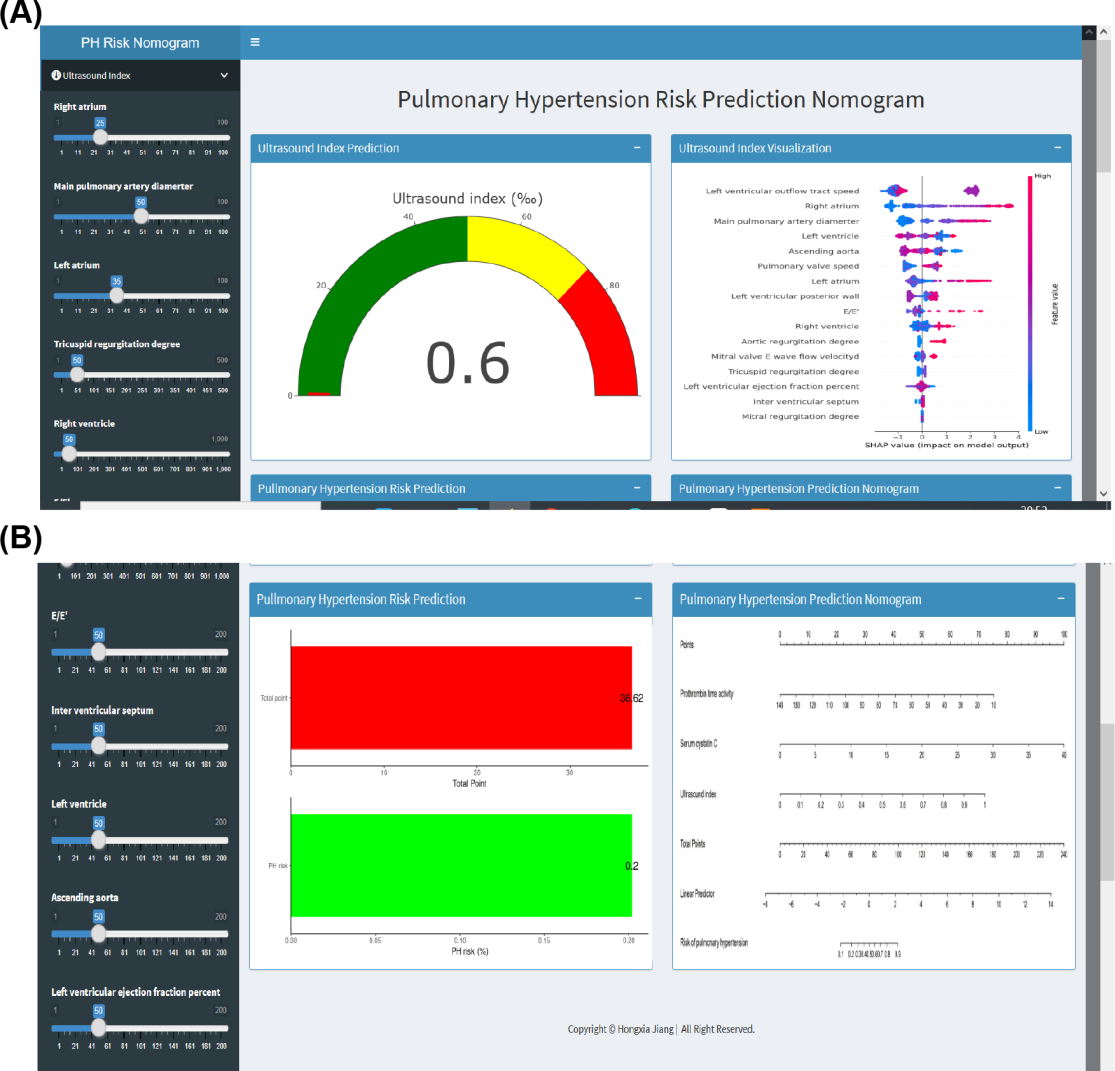
Screenshots of the nomogram web calculator. (A) Screenshot of the web calculator showing the calculated PH probability based on ultrasound index input and SHAP-based interpretation of feature importance for the ultrasound index variables. (B) Screenshot of the web calculator displaying the total points calculated via nomogram and the corresponding PH risk probability. E/E’: ratio of mitral valve early diastolic inflow velocity (E) to mitral annulus early diastolic velocity (E’); PH: pulmonary hypertension; SHAP: Shapley Additive Explanations.

To facilitate clinical implementation of our model, we developed a web-based calculator (Figure 5A and B), available online [16], which enables user-friendly PH risk assessment. This tool is expected to significantly enhance the model’s clinical adoption.

## Discussion

### Principal Findings

We present an integrated diagnostic model that combines ultrasound-derived parameters with clinical variables to improve early PH risk prediction. Using RFE, LASSO regression, and various machine learning algorithms, we optimized the model and rigorously assessed its predictive performance. Given that PH is a rapidly progressive disease with high mortality, early detection and intervention are essential for improving outcomes [17,18]. Current diagnostic methods for pulmonary artery pressure rely heavily on 2 approaches: RHC and echocardiography, which estimate pressure based on TRV [19-21]. However, catheterization is invasive, and echocardiography can be prone to significant inaccuracies. To overcome these limitations, our research proposes a novel noninvasive diagnostic pathway that integrates ultrasound and clinical data, thereby providing an effective tool for the early detection of PH.

In our model, 16 echocardiographic features (right atrium diameter, PAD, left atrium diameter, tricuspid valve reflux degree, right ventricular diameter, E/E’ [Ratio of Mitral Valve Early Diastolic Inflow Velocity (E) to Mitral Annulus Early Diastolic Velocity (E’)], interventricular septal thickness, left ventricular diameter, ascending aortic diameter, left ventricular ejection fraction, left ventricular outflow tract velocity, mitral valve reflux degree, pulmonary valve outflow velocity, mitral valve inflow velocity, aortic valve reflux degree, and left ventricular posterior wall thickness) and 2 laboratory tests variables (prothrombin time activity and CysC) was identified to be the critical predictive parameters of PH. These structural cardiac measurements demonstrate strong pathophysiological concordance with known PH mechanisms: enlargement of the right and left atria reflects increased pulmonary circulation pressure and heightened right ventricular load, both of which are common in patients with PH [22]. Additionally, an enlarged PAD correlates directly with elevated pulmonary vascular resistance, further exacerbating right ventricular pressure overload [23].

While the association between cardiac structural or functional parameters and PH is well-established and mechanistically straightforward, our selected biochemical markers (prothrombin time activity and CysC) similarly demonstrate previously reported—yet less widely recognized—pathophysiological links to PH development. A review summarizing the association between coagulation abnormalities and hypertension suggests that prothrombin time activity is closely correlated with elevated systolic and diastolic blood pressure in both hypertensive patients and normotensive individuals [24]. This is consistent with the conclusion in this paper that prothrombin time activity is associated with increased pulmonary artery pressure. CysC is generally recognized as an indicator of renal function. However, previous studies have found that CysC is also elevated in patients with pulmonary arterial hypertension and is positively correlated with right ventricular systolic pressure, right ventricular end-diastolic volume, and right ventricular end-systolic volume, suggesting its potential as a biomarker for PH [25].

Our study advances beyond prior research by systematically identifying optimal ultrasound parameters through rigorous RFE coupled with 10-fold cross-validation, ensuring robust and reproducible feature selection. Whereas conventional approaches typically analyze isolated echocardiographic measures, our novel methodology integrates multidimensional cardiac imaging features with critical clinical biomarkers to develop a high-performance yet clinically interpretable prediction model. This integrative approach demonstrates superior predictive accuracy for PH, representing a paradigm shift from single-parameter assessment to comprehensive risk stratification. The clinical implementation of this model enables: (1) earlier detection of subclinical PH through sensitive ultrasound biomarkers, (2) improved risk discrimination via combined imaging and laboratory data, and (3) actionable outputs for timely therapeutic decision-making.

It is also worth mentioning that in our study, we used multiple machine learning algorithms, such as XGBoost, random forest, and logistic regression. Among these models, XGBoost demonstrated the best predictive performance, achieving an AUC of 0.997, highlighting its strength in handling high-dimensional data and modeling complex nonlinear relationships. As a gradient-boosting algorithm, XGBoost incrementally reduces the model error to enhance predictive accuracy, rendering it particularly effective for clinical data with intricate interactions [26,27]. To enhance the interpretability of the XGBoost model, we constructed an analysis using SHAP. SHAP values quantified the contribution of each feature to the model’s predictions, emphasizing the importance of variables such as the right atrium and PAD in predicting PH. Compared to traditional “black-box” models, incorporating SHAP analysis significantly enhanced the model’s interpretability, providing more clinical insights. SHAP not only increases transparency but also enables clinicians to better understand the predictive mechanisms of the model, thus supporting more informed and reasoned clinical decision-making.

To enable seamless adoption in clinical workflows, we deployed the nomogram and risk calculator [16] as a web-based Shiny application with an Application Programming Interface compatible with major EHR systems. Compliant with Health Level Seven Fast Healthcare Interoperability Resources standards, the tool automatically retrieves echocardiographic measurements and laboratory values, computes the PH risk score in real time, and presents results in clinician-facing dashboards during outpatient visits. Moreover, the application can be embedded within ultrasound reporting software, allowing sonographers to generate risk estimates immediately at the point of image acquisition. Concrete examples of our predictive model’s clinical application are: a 58-year-old woman presenting with unexplained dyspnea underwent noninvasive PH risk stratification using our proposed nomogram or web-based tool to defer immediate RHC. The clinician input key echocardiographic parameters (right atrium diameter=4.5 cm, PAD=2.8 cm, left atrium diameter=4.0 cm, and other selected parameters in our model) along with laboratory values (prothrombin time activity=78%, serum CysC=1.5 mg/L) extracted from recent reports. Application of the nomogram yielded a total score of 50 points, corresponding to an 85% predicted PH risk, automatically flagging the patient as high-risk. This prompted clinical actions, including prioritization of confirmatory RHC, initiation of enhanced monitoring (repeat echocardiography and N-terminal pro-B-type Natriuretic Peptide testing, and consideration of early PH specialty referral. For seamless clinical implementation, the tool could integrate with electronic health record systems (eg, Cerner, formerly known as Cerner Corporation, now part of Oracle Health) as a plug-in module, enabling automated data population from EHR fields (laboratory results or echocardiographic reports) and generation of standardized risk assessment documents for longitudinal patient tracking within the medical record.

Although several machine learning models have been developed for PH, they exhibit certain limitations. Many models rely on a single data source, which restricts predictive accuracy and reliability. For instance, the study by Athénaïs Boucly identified cytokines as prognostic biomarkers in pulmonary arterial hypertension but failed to account for potential confounding factors, such as clinical variables or imaging data, which may influence the results [28]. Similarly, the research by Hirata et al [29] demonstrated improved accuracy of a machine learning model compared to traditional methods in the derivation cohort; however, its performance in the validation cohort was only comparable to guideline-based echocardiographic assessments, underscoring the need for further optimization and validation. Additionally, previous studies frequently used univariable and multivariable models to evaluate the relationship between clinical and echocardiographic parameters in precapillary PH [30]. However, they lacked advanced feature selection methods such as LASSO and RFE, which could have enhanced model performance by identifying key predictive variables, reducing overfitting, and providing deeper insights into prognostic factors. In contrast, our research proposes an innovative approach by integrating ultrasound parameters with clinical variables, thereby enhancing the model’s predictive performance through multidimensional integration [31,32]. Unlike prior work, we optimized feature selection using both RFE and LASSO regression and ensured model robustness through comparison and tuning across multiple machine-learning models [33]. We also significantly enhanced model interpretability using SHAP, which makes this high-performing “black-box” model more transparent and trustworthy for clinical use, ultimately enhancing its applicability and credibility in a health care context.

Despite the strong performance of our model in predicting PH, it exhibits certain limitations. First, the study sample size was relatively small, which limits the generalizability of the findings. Future work should involve validation using larger, multicenter datasets to ensure the model’s external validity. Second, as new ultrasound technologies and biomarkers emerge, future research should incorporate additional multidimensional biological data to enhance the predictive accuracy and applicability of the model.

### Conclusions

We developed a high-performance PH prediction model using machine learning analysis of echocardiographic, laboratory, and demographic data from 895 participants. The model incorporates 16 key ultrasound parameters and 2 biomarkers, with validation showing excellent accuracy. We also created a web-based calculator to facilitate clinical use, providing a practical tool for early, noninvasive PH detection.

## Supplementary material

10.2196/74117 Multimedia Appendix 1 Independent external validation dataset.

## Abbreviations

AUC: area under the receiver operating characteristic curve
CysC: cystatin C
DCA: decision curve analysis
E/E': ratio of mitral valve early diastolic inflow velocity (E) to mitral annulus early diastolic velocity (E')
EHR: electronic health record
LASSO: least absolute shrinkage and selection operator
mPAP: mean pulmonary artery pressure
PAD: pulmonary artery diameter
PH: pulmonary hypertension
RFE: recursive feature elimination
RHC: right heart catheterization
ROC: receiver operating characteristic
SHAP: Shapley Additive Explanations
TRV: tricuspid regurgitation velocity
XGBoost: Extreme Gradient Boosting
