# Supplementary material for: Interpretable Machine Learning Model for Pulmonary Hypertension Risk Prediction: Retrospective Cohort Study
Source: JMIR Med Inform. 2025 Sep 24;13:e74117. doi: 10.2196/74117 (PMC12459742; doi:10.2196/74117)
Supplement: Multimedia Appendix 1 [file medinform-v13-e74117-s001.docx]

**Table S1.** Independent external validation dataset.

| **Variables** | | **Normal (n=155)** | **PH^a^ (n=126)** | **Overall (n=281)** | **P value** |
| --- | --- | --- | --- | --- | --- |
| Age (year) | |  |  |  | <.001 |
|  | Mean (SD) | 44.8 (21.7) | 58.1 (22.6) | 50.8 (23.0) |  |
|  | Median (Min^b^, Max)^c^ | 51.0 (0, 97.0) | 62.5 (3.00, 95.0) | 55.0 (0, 97.0) |  |
| Sex, n (%) | |  |  |  | 1 |
|  | Male | 87 (56.1) | 71 (56.3) | 158 (56.2) |  |
|  | Female | 68 (43.9) | 55 (43.7) | 123 (43.8) |  |
| Ascending aortic diameter (AAD, cm) | |  |  |  | <.001 |
|  | Mean (SD) | 2.81 (0.700) | 3.17 (0.613) | 2.97 (0.685) |  |
|  | Median (Min, Max) | 3.00 (0.700, 4.30) | 3.20 (1.20, 5.10) | 3.10(0.700, 5.10) |  |
| Left atrium diameter (LAD, cm) | |  |  |  | <.001 |
|  | Mean (SD) | 3.04 (0.751) | 3.99 (1.10) | 3.47 (1.03) |  |
|  | Median (Min, Max) | 3.10 (0.800, 5.30) | 3.90 (2.00, 9.10) | 3.40(0.800, 9.10) |  |
| Left ventricular diameter (LVD, cm) | |  |  |  | <.001 |
|  | Mean (SD) | 4.15 (0.863) | 4.54 (0.992) | 4.33 (0.942) |  |
|  | Median (Min, Max) | 4.30 (1.20, 5.60) | 4.40 (2.70, 8.00) | 4.30 (1.20, 8.00) |  |
| Interventricular septal thickness (IVS, cm) | |  |  |  | <.001 |
|  | Mean (SD) | 0.903 (0.223) | 1.04 (0.208) | 0.963 (0.226) |  |
|  | Median (Min, Max) | 0.900 (0.300, 1.50) | 1.00 (0.500, 1.80) | 1.00(0.300, 1.80) |  |
|  | Missing, n (%) | 0 (0) | 2 (1.6) | 2 (0.7) |  |
| Left ventricular posterior wall thickness (LVPW, cm) | |  |  |  | <.001 |
|  | Mean (SD) | 0.879 (0.207) | 0.986 (0.184) | 0.927 (0.204) |  |
|  | Median (Min, Max) | 0.900 (0.300, 1.20) | 1.00 (0.500, 1.40) | 1.00(0.300, 1.40) |  |
|  | Missing, n (%) | 2 (1.3) | 2 (1.6) | 4 (1.4) |  |
| Right atrium diameter (RAD, cm) | |  |  |  | <.001 |
|  | Mean (SD) | 3.13 (0.688) | 4.07 (0.824) | 3.55 (0.882) |  |
|  | Median (Min, Max) | 3.30 (1.10, 5.00) | 3.90 (2.30, 6.60) | 3.50 (1.10, 6.60) |  |
|  | Missing, n (%) | 0 (0) | 1 (0.8) | 1 (0.4) |  |
| Right ventricular diameter (RVD, cm) | |  |  |  | <.001 |
|  | Mean (SD) | 2.93 (0.645) | 3.67 (0.815) | 3.26 (0.813) |  |
|  | Median (Min, Max) | 3.00 (1.00, 4.90) | 3.50 (2.20, 6.80) | 3.20 (1.00, 6.80) |  |
|  | Missing, n (%) | 1 (0.6) | 0 (0) | 1 (0.4) |  |
| Pulmonary artery diameter (PAD, cm) | |  |  |  | <.001 |
|  | Mean (SD) | 2.05 (0.467) | 2.64 (0.653) | 2.32 (0.630) |  |
|  | Median (Min, Max) | 2.10 (0.500, 3.00) | 2.50 (1.40, 5.30) | 2.30(0.500, 5.30) |  |
|  | Missing, n (%) | 3 (1.9) | 0 (0) | 3 (1.1) |  |
| Left ventricular fractional shortening (LVFS, %) | |  |  |  | .161 |
|  | Mean (SD) | 34.3 (3.30) | 33.4 (6.04) | 33.9 (4.69) |  |
|  | Median (Min, Max) | 35.0 (27.0, 43.0) | 35.0 (11.0, 47.0) | 35.0 (11.0, 47.0) |  |
|  | Missing, n (%) | 2 (1.3) | 11 (8.7) | 13 (4.6) |  |
| Left ventricular ejection fraction (LVEF, %) | |  |  |  | .003 |
|  | Mean (SD) | 64.0 (4.40) | 60.8 (11.3) | 62.6 (8.38) |  |
|  | Median (Min, Max) | 65.0 (41.0, 75.0) | 63.0 (19.0, 79.0) | 65.0 (19.0, 79.0) |  |
|  | Missing, n (%) | 0 (0) | 1 (0.8) | 1 (0.4) |  |
| Mitral valve inflow velocity (MV Vmax, m/s) | |  |  |  | <.001 |
|  | Mean (SD) | 0.765 (0.209) | 0.923 (0.361) | 0.837 (0.298) |  |
|  | Median (Min, Max) | 0.700 (0.400, 1.30) | 0.900 (0.400, 2.90) | 0.800(0.400,2.90) |  |
|  | Missing, n (%) | 5 (3.2) | 1 (0.8) | 6 (2.1) |  |
| Mitral inflow A-wave peak velocity (MPAV, m/s) | |  |  |  | .447 |
|  | Mean (SD) | 0.765 (0.200) | 0.790 (0.291) | 0.775 (0.242) |  |
|  | Median (Min, Max) | 0.800 (0.400, 1.50) | 0.800 (0.300, 1.70) | 0.800(0.300, 1.70) |  |
|  | Missing, n (%) | 10 (6.5) | 19 (15.1) | 29 (10.3) |  |
| Left ventricular outflow tract velocity (LVOT, m/s) | |  |  |  | .537 |
|  | Mean (SD) | 0.924 (0.243) | 1.03 (0.821) | 0.974 (0.597) |  |
|  | Median (Min, Max) | 0.900 (0.600, 1.70) | 0.800 (0.300, 4.70) | 0.900(0.300, 4.70) |  |
|  | Missing, n (%) | 126 (81.3) | 98 (77.8) | 224 (79.7) |  |
| Aortic valve outflow velocity (AV Vmax, m/s) | |  |  |  | .01 |
|  | Mean (SD) | 1.25 (0.295) | 1.38 (0.445) | 1.31 (0.374) |  |
|  | Median (Min, Max) | 1.20 (0.700, 2.70) | 1.30 (0.500, 3.50) | 1.30 (0.500, 3.50) |  |
|  | Missing, n (%) | 3 (1.9) | 3 (2.4) | 6 (2.1) |  |
| Pulmonary valve outflow velocity (PV Vmax, m/s) | |  |  |  | .298 |
|  | Mean (SD) | 0.954 (0.187) | 0.985 (0.258) | 0.967 (0.220) |  |
|  | Median (Min, Max) | 0.900 (0.600, 1.90) | 1.00 (0.500, 1.90) | 0.950(0.500, 1.90) |  |
|  | Missing, n (%) | 11 (7.1) | 16 (12.7) | 27 (9.6) |  |
| Mitral valve reflux degree (MVRD, 0-3) | |  |  |  | <.001 |
|  | Mean (SD) | 0.374 (0.508) | 1.12 (0.924) | 0.708 (0.813) |  |
|  | Median (Min, Max) | 0 (0, 2.00) | 1.00 (0, 3.00) | 1.00 (0, 3.00) |  |
| Aortic valve reflux degree (AVRD, 0-3) | |  |  |  | <.001 |
|  | Mean (SD) | 0.232 (0.553) | 0.603 (0.658) | 0.399 (0.629) |  |
|  | Median (Min, Max) | 0 (0, 3.00) | 1.00 (0, 3.00) | 0 (0, 3.00) |  |
| Tricuspid valve reflux degree (TVRD, 0-3) | |  |  |  | <.001 |
|  | Mean (SD) | 0.561 (0.536) | 1.65 (0.865) | 1.05 (0.886) |  |
|  | Median (Min, Max) | 1.00 (0, 2.00) | 1.50 (0, 4.00) | 1.00 (0, 4.00) |  |
| Widening of ascending aorta, n (%) | |  |  |  | <.001 |
|  | No | 155 (100) | 93 (73.8) | 248 (88.3) |  |
|  | Yes | 0 (0) | 33 (26.2) | 33 (11.7) |  |
| Aortic valve thickening, n (%) | |  |  |  | <.001 |
|  | No | 155 (100) | 98 (77.8) | 253 (90) |  |
|  | Yes | 0 (0) | 28 (22.2) | 28 (10) |  |
| Aortic valve echo intensification, n (%) | |  |  |  | <.001 |
|  | No | 155 (100) | 99 (78.6) | 254 (90.4) |  |
|  | Yes | 0 (0) | 27 (21.4) | 27 (9.6) |  |
| Aortic valve calcification, n (%) | |  |  |  | .005 |
|  | No | 155 (100) | 118 (93.7) | 273 (97.2) |  |
|  | Yes | 0 (0) | 8 (6.3) | 8 (2.8) |  |
| Poor closure of the aortic valve, n (%) | |  |  |  | <.001 |
|  | No | 155 (100) | 74 (58.7) | 229 (81.5) |  |
|  | Yes | 0 (0) | 52 (41.3) | 52 (18.5) |  |
| Pulmonary artery widening, n (%) | |  |  |  | <.001 |
|  | No | 155 (100) | 96 (76.2) | 251 (89.3) |  |
|  | Yes | 0 (0) | 30 (23.8) | 30 (10.7) |  |
| Poor closure of the pulmonary valve, n (%) | |  |  |  | .002 |
|  | No | 155 (100) | 117 (92.9) | 272 (96.8) |  |
|  | Yes | 0 (0) | 9 (7.1) | 9 (3.2) |  |
| Mitral valve insufficiency, n (%) | |  |  |  | <.001 |
|  | No | 155 (100) | 52 (41.3) | 207 (73.7) |  |
|  | Yes | 0 (0) | 74 (58.7) | 74 (26.3) |  |
| Tricuspid insufficiency, n (%) | |  |  |  | <.001 |
|  | No | 155 (100) | 17 (13.5) | 172 (61.2) |  |
|  | Yes | 0 (0) | 109 (86.5) | 109 (38.8) |  |
| Ventricular septum thickened, n (%) | |  |  |  | <.001 |
|  | No | 155 (100) | 93 (73.8) | 248 (88.3) |  |
|  | Yes | 0 (0) | 33 (26.2) | 33 (11.7) |  |
| Left ventricular posterior wall thickened, n (%) | |  |  |  | <.001 |
|  | No | 155 (100) | 109 (86.5) | 264 (94) |  |
|  | Yes | 0 (0) | 17 (13.5) | 17 (6) |  |
| Atrial septal defect, n (%) | |  |  |  | .002 |
|  | No | 155 (100) | 117 (92.9) | 272 (96.8) |  |
|  | Yes | 0 (0) | 9 (7.1) | 9 (3.2) |  |
| Ventricular septal defect, n (%) | |  |  |  | .084 |
|  | No | 155 (100) | 122 (96.8) | 277 (98.6) |  |
|  | Yes | 0 (0) | 4 (3.2) | 4 (1.4) |  |
| E/E’^d^ | |  |  |  | <.001 |
|  | Mean (SD) | 9.54 (2.68) | 13.4 (5.37) | 11.5 (4.67) |  |
|  | Median (Min, Max) | 10.0 (3.00, 20.0) | 12.0 (4.00, 30.0) | 10.0 (3.00, 30.0) |  |
|  | Missing, n (%) | 42 (27.1) | 11 (8.7) | 53 (18.9) |  |
| WBC^e^ (10^9^/L) | |  |  |  | .012 |
|  | Mean (SD) | 8.14 (4.61) | 6.93 (3.24) | 7.59 (4.08) |  |
|  | Median (Min, Max) | 6.80 (1.99, 30.6) | 6.07 (2.20, 22.1) | 6.44 (1.99, 30.6) |  |
|  | Missing, n (%) | 7 (4.5) | 2 (1.6) | 9 (3.2) |  |
| RBC^f^ (10^9^/L) | |  |  |  | .487 |
|  | Mean (SD) | 3.98 (0.829) | 3.91 (0.813) | 3.95 (0.821) |  |
|  | Median (Min, Max) | 4.10 (1.65, 5.62) | 3.83 (2.01, 6.10) | 3.99 (1.65, 6.10) |  |
|  | Missing, n (%) | 7 (4.5) | 2 (1.6) | 9 (3.2) |  |
| Hb^g^ (g/L) | |  |  |  | .131 |
|  | Mean (SD) | 123 (24.8) | 118 (26.7) | 121 (25.8) |  |
|  | Median (Min, Max) | 126 (58.0, 169) | 118 (55.7, 199) | 122 (55.7, 199) |  |
|  | Missing, n (%) | 7 (4.5) | 2 (1.6) | 9 (3.2) |  |
| Plt^h^ (10^9^/L) | |  |  |  | .002 |
|  | Mean (SD) | 216 (87.1) | 183 (89.5) | 201 (89.6) |  |
|  | Median (Min, Max) | 208 (35.0, 506) | 161 (51.0, 599) | 185 (35.0, 599) |  |
|  | Missing, n (%) | 7 (4.5) | 2 (1.6) | 9 (3.2) |  |
| Neutrophilic granulocyte percentage (%) | |  |  |  | .044 |
|  | Mean (SD) | 65.1 (16.1) | 68.7 (12.8) | 66.7 (14.8) |  |
|  | Median (Min, Max) | 65.9 (14.5, 95.5) | 67.2 (37.4, 96.7) | 66.9 (14.5, 96.7) |  |
|  | Missing, n (%) | 7 (4.5) | 2 (1.6) | 9 (3.2) |  |
| Lymphocytes percentage (%) | |  |  |  | .042 |
|  | Mean (SD) | 23.5 (14.1) | 20.5 (10.3) | 22.1 (12.6) |  |
|  | Median (Min, Max) | 22.2 (1.30, 81.2) | 20.2 (1.20, 47.2) | 21.1 (1.20, 81.2) |  |
|  | Missing, n (%) | 7 (4.5) | 2 (1.6) | 9 (3.2) |  |
| Monocyte percentage (%) | |  |  |  | .533 |
|  | Mean (SD) | 8.43 (5.49) | 8.10 (3.03) | 8.28 (4.53) |  |
|  | Median (Min, Max) | 7.30 (0.500, 61.2) | 8.10 (1.50, 20.5) | 7.60 (0.500, 61.2) |  |
|  | Missing, n (%) | 7 (4.5) | 2 (1.6) | 9 (3.2) |  |
| Eosinophils percentage (%) | |  |  |  | .193 |
|  | Mean (SD) | 2.47 (3.67) | 2.01 (2.02) | 2.26 (3.03) |  |
|  | Median (Min, Max) | 1.50 (0, 31.2) | 1.40 (0, 10.2) | 1.50 (0, 31.2) |  |
|  | Missing, n (%) | 7 (4.5) | 2 (1.6) | 9 (3.2) |  |
| Basophils percentage (%) | |  |  |  | .197 |
|  | Mean (SD) | 0.486 (0.326) | 0.764 (2.37) | 0.613 (1.62) |  |
|  | Median (Min, Max) | 0.400 (0, 1.40) | 0.500 (0, 26.5) | 0.500 (0, 26.5) |  |
|  | Missing, n (%) | 7 (4.5) | 2 (1.6) | 9 (3.2) |  |
| Neutrophil absolute value (10^9^/L) | |  |  |  | .253 |
|  | Mean (SD) | 5.47 (3.72) | 4.99 (3.17) | 5.25 (3.48) |  |
|  | Median (Min, Max) | 4.23 (0.400, 22.4) | 3.72 (1.45, 20.0) | 4.00 (0.400, 22.4) |  |
|  | Missing, n (%) | 7 (4.5) | 2 (1.6) | 9 (3.2) |  |
| Lymphocyte absolute value (10^9^/L) | |  |  |  | <.001 |
|  | Mean (SD) | 1.67 (1.07) | 1.25 (0.671) | 1.48 (0.931) |  |
|  | Median (Min, Max) | 1.50 (0.0400, 6.27) | 1.20 (0.130, 4.70) | 1.30(0.0400, 6.27) |  |
|  | Missing, n (%) | 7 (4.5) | 2 (1.6) | 9 (3.2) |  |
| Monocyte absolute value (10^9^/L) | |  |  |  | .046 |
|  | Mean (SD) | 0.733 (1.31) | 0.513 (0.220) | 0.633 (0.983) |  |
|  | Median (Min, Max) | 0.500 (0, 15.6) | 0.500 (0.150, 1.23) | 0.500 (0, 15.6) |  |
|  | Missing, n (%) | 7 (4.5) | 2 (1.6) | 9 (3.2) |  |
| Eosinophils absolute value (10^9^/L) | |  |  |  | .088 |
|  | Mean (SD) | 0.237 (0.827) | 0.119 (0.124) | 0.183 (0.617) |  |
|  | Median (Min, Max) | 0.100 (0, 9.55) | 0.100 (0, 0.660) | 0.100 (0, 9.55) |  |
|  | Missing, n (%) | 7 (4.5) | 2 (1.6) | 9 (3.2) |  |
| Basophil absolute value (10^9^/L) | |  |  |  | .392 |
|  | Mean (SD) | 0.0259 (0.0415) | 0.0403 (0.183) | 0.0325 (0.127) |  |
|  | Median (Min, Max) | 0.0100 (0, 0.250) | 0.0100 (0, 2.02) | 0.0100 (0, 2.02) |  |
|  | Missing, n (%) | 7 (4.5) | 2 (1.6) | 9 (3.2) |  |
| HCT^i^ (%) | |  |  |  | .265 |
|  | Mean (SD) | 36.6 (7.17) | 35.6 (7.70) | 36.1 (7.42) |  |
|  | Median (Min, Max) | 37.7 (17.3, 50.5) | 35.4 (19.1, 56.6) | 36.5 (17.3, 56.6) |  |
|  | Missing, n (%) | 7 (4.5) | 2 (1.6) | 9 (3.2) |  |
| Mean RBC volume (fL) | |  |  |  | .127 |
|  | Mean (SD) | 92.6 (8.23) | 91.1 (7.50) | 91.9 (7.93) |  |
|  | Median (Min, Max) | 91.8 (60.2, 116) | 91.7 (60.2, 111) | 91.7 (60.2, 116) |  |
|  | Missing, n (%) | 7 (4.5) | 2 (1.6) | 9 (3.2) |  |
| Average hemoglobin content (pg) | |  |  |  | .019 |
|  | Mean (SD) | 31.1 (3.18) | 30.2 (2.95) | 30.7 (3.10) |  |
|  | Median (Min, Max) | 30.9 (18.1, 39.1) | 30.5 (17.1, 37.3) | 30.7 (17.1, 39.1) |  |
|  | Missing, n (%) | 7 (4.5) | 2 (1.6) | 9 (3.2) |  |
| Average hemoglobin concentration (g/L) | |  |  |  | .004 |
|  | Mean (SD) | 336 (11.3) | 331 (12.7) | 334 (12.1) |  |
|  | Median (Min, Max) | 336 (299, 393) | 333 (285, 355) | 335 (285, 393) |  |
|  | Missing, n (%) | 7 (4.5) | 2 (1.6) | 9 (3.2) |  |
| Erythrocyte distribution width CV^j^ (%) | |  |  |  | .047 |
|  | Mean (SD) | 14.7 (2.59) | 15.5 (3.74) | 15.0 (3.19) |  |
|  | Median (Min, Max) | 13.7 (11.5, 26.2) | 14.3 (11.9, 33.1) | 14.0 (11.5, 33.1) |  |
|  | Missing, n (%) | 8 (5.2) | 2 (1.6) | 10 (3.6) |  |
| Mean platelet volume (fL) | |  |  |  | .867 |
|  | Mean (SD) | 8.89 (1.43) | 8.86 (1.38) | 8.88 (1.40) |  |
|  | Median (Min, Max) | 8.70 (6.30, 15.1) | 8.55 (6.50, 12.6) | 8.70 (6.30, 15.1) |  |
|  | Missing, n (%) | 7 (4.5) | 2 (1.6) | 9 (3.2) |  |
| PT^k^ (s) | |  |  |  | .02 |
|  | Mean (SD) | 12.3 (1.97) | 13.0 (3.06) | 12.6 (2.55) |  |
|  | Median (Min, Max) | 11.8 (8.50, 22.2) | 12.3 (9.90, 31.2) | 12.0 (8.50, 31.2) |  |
|  | Missing, n (%) | 6 (3.9) | 2 (1.6) | 8 (2.8) |  |
| INR^l^ | |  |  |  | .018 |
|  | Mean (SD) | 1.13 (0.189) | 1.21 (0.297) | 1.17 (0.246) |  |
|  | Median (Min, Max) | 1.09 (0.770, 2.10) | 1.14 (0.900, 2.99) | 1.11 (0.770, 2.99) |  |
|  | Missing, n (%) | 6 (3.9) | 2 (1.6) | 8 (2.8) |  |
| Prothrombin time activity (%) | |  |  |  | .009 |
|  | Mean (SD) | 86.4 (15.9) | 81.0 (17.7) | 84.0 (16.9) |  |
|  | Median (Min, Max) | 88.0 (35.0, 145) | 82.0 (23.0, 118) | 85.0 (23.0, 145) |  |
|  | Missing, n (%) | 6 (3.9) | 2 (1.6) | 8 (2.8) |  |
| APTT^m^ (s) | |  |  |  | .029 |
|  | Mean (SD) | 34.1 (10.4) | 32.0 (4.48) | 33.2 (8.29) |  |
|  | Median (Min, Max) | 31.1 (21.8, 83.4) | 31.5 (22.4, 45.0) | 31.2 (21.8, 83.4) |  |
|  | Missing, n (%) | 6 (3.9) | 2 (1.6) | 8 (2.8) |  |
| TT^n^ (s) | |  |  |  | .745 |
|  | Mean (SD) | 15.5 (5.98) | 15.3 (3.97) | 15.4 (5.16) |  |
|  | Median (Min, Max) | 14.2 (10.7, 55.9) | 14.8 (11.6, 55.0) | 14.4 (10.7, 55.9) |  |
|  | Missing, n (%) | 6 (3.9) | 2 (1.6) | 8 (2.8) |  |
| Fibrinogen content (mg/dL) | |  |  |  | .806 |
|  | Mean (SD) | 325 (131) | 321 (119) | 323 (126) |  |
|  | Median (Min, Max) | 303 (24.0, 926) | 290 (141, 777) | 297 (24.0, 926) |  |
|  | Missing, n (%) | 6 (3.9) | 2 (1.6) | 8 (2.8) |  |
| D2^o^ dimer (ng/mL) | |  |  |  | .393 |
|  | Mean (SD) | 1970 (3560) | 1620 (2590) | 1810 (3160) |  |
|  | Median (Min, Max) | 584 (68.0, 20800) | 685 (60.0, 17700) | 646 (60.0, 20800) |  |
|  | Missing, n (%) | 31 (20) | 25 (19.8) | 56 (19.9) |  |
| ALT^p^ (U/L) | |  |  |  | .015 |
|  | Mean (SD) | 66.2 (172) | 30.3 (43.3) | 50.0 (132) |  |
|  | Median (Min, Max) | 21.0 (1.00, 1210) | 17.0 (2.00, 322) | 19.0 (1.00, 1210) |  |
|  | Missing, n (%) | 7 (4.5) | 5 (4) | 12 (4.3) |  |
| AST^q^ (U/L) | |  |  |  | .028 |
|  | Mean (SD) | 76.0 (226) | 34.3 (33.9) | 57.2 (170) |  |
|  | Median (Min, Max) | 25.0 (5.00, 1920) | 22.0 (8.00, 210) | 24.0 (5.00, 1920) |  |
|  | Missing, n (%) | 6 (3.9) | 4 (3.2) | 10 (3.6) |  |
| AST/ALT | |  |  |  | .499 |
|  | Mean (SD) | 1.64 (1.71) | 1.78 (1.67) | 1.71 (1.69) |  |
|  | Median (Min, Max) | 1.23 (0.140, 16.0) | 1.35 (0.130, 13.7) | 1.31 (0.130, 16.0) |  |
|  | Missing, n (%) | 7 (4.5) | 5 (4) | 12 (4.3) |  |
| TBIL^r^ (μmol/L) | |  |  |  | .007 |
|  | Mean (SD) | 23.1 (33.5) | 15.3 (10.5) | 19.6 (26.1) |  |
|  | Median (Min, Max) | 12.6 (3.00, 239) | 12.6 (3.60, 73.0) | 12.6 (3.00, 239) |  |
|  | Missing, n (%) | 6 (3.9) | 4 (3.2) | 10 (3.6) |  |
| DBIL^s^ (μmol/L) | |  |  |  | .437 |
|  | Mean (SD) | 8.53 (15.0) | 9.61 (6.94) | 9.02 (12.0) |  |
|  | Median (Min, Max) | 4.20 (0.200, 99.2) | 7.85 (1.90, 62.8) | 6.10 (0.200, 99.2) |  |
|  | Missing, n (%) | 6 (3.9) | 4 (3.2) | 10 (3.6) |  |
| IDBIL^t^ (μmol/L) | |  |  |  | <.001 |
|  | Mean (SD) | 14.6 (26.0) | 5.67 (5.00) | 10.6 (20.0) |  |
|  | Median (Min, Max) | 7.60 (0.600, 228) | 4.50 (0.700, 40.0) | 6.20 (0.600, 228) |  |
|  | Missing, n (%) | 6 (3.9) | 4 (3.2) | 10 (3.6) |  |
| Total protein (g/L) | |  |  |  | .435 |
|  | Mean (SD) | 66.6 (9.21) | 65.8 (7.69) | 66.3 (8.56) |  |
|  | Median (Min, Max) | 68.0 (42.4, 85.7) | 65.6 (45.9, 90.3) | 66.7 (42.4, 90.3) |  |
|  | Missing, n (%) | 6 (3.9) | 4 (3.2) | 10 (3.6) |  |
| Alb^u^ (g/L) | |  |  |  | .124 |
|  | Mean (SD) | 40.0 (5.63) | 39.0 (4.98) | 39.5 (5.36) |  |
|  | Median (Min, Max) | 40.4 (26.9, 50.9) | 38.3 (25.5, 55.0) | 39.6 (25.5, 55.0) |  |
|  | Missing, n (%) | 6 (3.9) | 4 (3.2) | 10 (3.6) |  |
| Glb^v^ (g/L) | |  |  |  | <.001 |
|  | Mean (SD) | 26.6 (6.30) | 39.0 (4.98) | 32.2 (8.42) |  |
|  | Median (Min, Max) | 26.9 (10.5, 49.7) | 38.3 (25.5, 55.0) | 32.2 (10.5, 55.0) |  |
|  | Missing, n (%) | 6 (3.9) | 4 (3.2) | 10 (3.6) |  |
| Alb/Glb | |  |  |  | .064 |
|  | Mean (SD) | 1.59 (0.440) | 1.50 (0.320) | 1.55 (0.392) |  |
|  | Median (Min, Max) | 1.56 (0.700, 3.44) | 1.49 (0.520, 2.47) | 1.54 (0.520, 3.44) |  |
|  | Missing, n (%) | 6 (3.9) | 4 (3.2) | 10 (3.6) |  |
| γGGT^w^ (U/L) | |  |  |  | .218 |
|  | Mean (SD) | 67.1 (104) | 53.1 (83.4) | 60.8 (95.1) |  |
|  | Median (Min, Max) | 31.0 (2.00, 647) | 25.0 (3.00, 619) | 26.0 (2.00, 647) |  |
|  | Missing, n (%) | 6 (3.9) | 4 (3.2) | 10 (3.6) |  |
| ALP^x^ (U/L) | |  |  |  | .102 |
|  | Mean (SD) | 118 (115) | 97.7 (83.2) | 109 (102) |  |
|  | Median (Min, Max) | 80.0 (27.0, 993) | 74.0 (24.0, 733) | 77.0 (24.0, 993) |  |
|  | Missing, n (%) | 6 (3.9) | 4 (3.2) | 10 (3.6) |  |
| TBA^y^ (μmol/L) | |  |  |  | .867 |
|  | Mean (SD) | 7.72 (18.1) | 8.04 (12.7) | 7.87 (15.8) |  |
|  | Median (Min, Max) | 2.95 (0.300, 139) | 4.30 (0.500, 89.0) | 3.35 (0.300, 139) |  |
|  | Missing, n (%) | 9 (5.8) | 4 (3.2) | 13 (4.6) |  |
| BUN^z^ (μmol/L) | |  |  |  | .002 |
|  | Mean (SD) | 6.11 (4.94) | 7.87 (4.15) | 6.91 (4.67) |  |
|  | Median (Min, Max) | 5.00 (0.700, 40.9) | 7.01 (3.25, 24.5) | 5.56 (0.700, 40.9) |  |
|  | Missing, n (%) | 6 (3.9) | 3 (2.4) | 9 (3.2) |  |
| Creatinine (μmol/L) | |  |  |  | .086 |
|  | Mean (SD) | 90.0 (153) | 124 (166) | 105 (160) |  |
|  | Median (Min, Max) | 63.4 (5.30, 1650) | 78.5 (17.0, 1150) | 70.0 (5.30, 1650) |  |
|  | Missing, n (%) | 6 (3.9) | 3 (2.4) | 9 (3.2) |  |
| Uric acid (μmol/L) | |  |  |  | .007 |
|  | Mean (SD) | 295 (112) | 334 (123) | 313 (119) |  |
|  | Median (Min, Max) | 289 (69.4, 774) | 335 (99.3, 709) | 307 (69.4, 774) |  |
|  | Missing, n (%) | 6 (3.9) | 3 (2.4) | 9 (3.2) |  |
| Carbon dioxide (mmol/L) | |  |  |  | .22 |
|  | Mean (SD) | 21.9 (3.64) | 22.5 (4.24) | 22.2 (3.93) |  |
|  | Median (Min, Max) | 22.0 (10.5, 35.2) | 21.9 (13.0, 44.7) | 22.0 (10.5, 44.7) |  |
|  | Missing, n (%) | 6 (3.9) | 3 (2.4) | 9 (3.2) |  |
| Serum cystatin C (mg/L) | |  |  |  | .016 |
|  | Mean (SD) | 1.42 (1.04) | 1.82 (1.57) | 1.60 (1.32) |  |
|  | Median (Min, Max) | 1.05 (0.360, 5.88) | 1.22 (0.600, 7.69) | 1.12 (0.360, 7.69) |  |
|  | Missing |  |  |  |  |
| Potassium (mmol/L) | |  |  |  | .27 |
|  | Mean (SD) | 4.03 (0.539) | 3.97 (0.488) | 4.00 (0.517) |  |
|  | Median (Min, Max) | 3.99 (3.01, 6.39) | 3.92 (2.68, 5.57) | 3.95 (2.68, 6.39) |  |
|  | Missing, n (%) | 9 (5.8) | 4 (3.2) | 13 (4.6) |  |
| Sodium (mmol/L) | |  |  |  | .331 |
|  | Mean (SD) | 139 (3.44) | 139 (3.18) | 139 (3.32) |  |
|  | Median (Min, Max) | 139 (127, 152) | 140 (129, 148) | 139 (127, 152) |  |
|  | Missing, n (%) | 9 (5.8) | 4 (3.2) | 13 (4.6) |  |
| Chlorine (mmol/L) | |  |  |  | .878 |
|  | Mean (SD) | 103 (4.09) | 103 (4.48) | 103 (4.26) |  |
|  | Median (Min, Max) | 103 (90.0, 114) | 104 (92.5, 114) | 103 (90.0, 114) |  |
|  | Missing, n (%) | 9 (5.8) | 4 (3.2) | 13 (4.6) |  |
| Calcium (mmol/L) | |  |  |  | .262 |
|  | Mean (SD) | 2.22 (0.154) | 2.20 (0.185) | 2.21 (0.169) |  |
|  | Median (Min, Max) | 2.24 (1.80, 2.75) | 2.18 (1.84, 3.12) | 2.20 (1.80, 3.12) |  |
|  | Missing, n (%) | 9 (5.8) | 4 (3.2) | 13 (4.6) |  |
| Magnesium (mmol/L) | |  |  |  | .789 |
|  | Mean (SD) | 0.875 (0.138) | 0.871 (0.119) | 0.873 (0.130) |  |
|  | Median (Min, Max) | 0.880 (0.570, 1.59) | 0.880 (0.520, 1.42) | 0.880(0.520, 1.59) |  |
|  | Missing, n (%) | 13 (8.4) | 8 (6.3) | 21 (7.5) |  |
| Phosphorus (mmol/L) | |  |  |  | .677 |
|  | Mean (SD) | 1.20 (0.364) | 1.18 (0.295) | 1.19 (0.334) |  |
|  | Median (Min, Max) | 1.15 (0.190, 2.24) | 1.14 (0.520, 2.14) | 1.14 (0.190, 2.24) |  |
|  | Missing, n (%) | 9 (5.8) | 4 (3.2) | 13 (4.6) |  |

**Notes:** ^a^PH: Pulmonary Hypertension.^b^Min: Minimum.^c^Max: Maximum.^d^E/E’: Ratio of Mitral Valve Early Diastolic Inflow Velocity (E) to Mitral Annulus Early Diastolic Velocity (E')^e^WBC: White Blood Cell^f^RBC: Red Blood Cell^g^Hb: Hemoglobin^h^Plt: Platelet count^i^HCT: Hematocrit^j^CV: Coefficient of Variation^k^PT: Prolonged Prothrombin Time.^l^INR: International Normalized Ratio.^m^APTT: Activated Partial Thromboplastin Time^n^TT: Thrombin Time^o^D2: D-Dimer^p^ALT: Alanine Aminotransferase^q^AST: Aspartate Aminotransferase^r^TBIL: Total Bilirubin^s^DBIL: Direct Bilirubin^t^IDBIL: Indirect Bilirubin^u^Alb: Albumin^v^Glb: Globulin^w^γGGT: Gamma-Glutamyl Transferase^x^ALP: Alkaline Phosphatase^y^TBA: Total Bile Acids^z^BUN: Blood Urea Nitrogen
